# Artemisinin-Naphthoquine versus Artemether-Lumefantrine for Uncomplicated Malaria in Papua New Guinean Children: An Open-Label Randomized Trial

**DOI:** 10.1371/journal.pmed.1001773

**Published:** 2014-12-30

**Authors:** Moses Laman, Brioni R. Moore, John M. Benjamin, Gumul Yadi, Cathy Bona, Jonathan Warrel, Johanna H. Kattenberg, Tamarah Koleala, Laurens Manning, Bernadine Kasian, Leanne J. Robinson, Naomi Sambale, Lina Lorry, Stephan Karl, Wendy A. Davis, Anna Rosanas-Urgell, Ivo Mueller, Peter M. Siba, Inoni Betuela, Timothy M. E. Davis

**Affiliations:** 1 School of Medicine and Pharmacology, University of Western Australia, Fremantle Hospital, Fremantle, Western Australia, Australia; 2 Papua New Guinea Institute of Medical Research, Madang, Madang Province, Papua New Guinea; 3 Infection and Immunity Division, Walter and Eliza Hall Institute, Parkville, Victoria, Australia; 4 Center de Recerca en Salut Internacional de Barcelona, Barcelona, Spain; Mahidol-Oxford Tropical Medicine Research Unit, Thailand

## Abstract

**Background:**

Artemisinin combination therapies (ACTs) with broad efficacy are needed where multiple *Plasmodium* species are transmitted, especially in children, who bear the brunt of infection in endemic areas. In Papua New Guinea (PNG), artemether-lumefantrine is the first-line treatment for uncomplicated malaria, but it has limited efficacy against *P. vivax*. Artemisinin-naphthoquine should have greater activity in vivax malaria because the elimination of naphthoquine is slower than that of lumefantrine. In this study, the efficacy, tolerability, and safety of these ACTs were assessed in PNG children aged 0.5–5 y.

**Methods and Findings:**

An open-label, randomized, parallel-group trial of artemether-lumefantrine (six doses over 3 d) and artemisinin-naphthoquine (three daily doses) was conducted between 28 March 2011 and 22 April 2013. Parasitologic outcomes were assessed without knowledge of treatment allocation. Primary endpoints were the 42-d *P. falciparum* PCR-corrected adequate clinical and parasitologic response (ACPR) and the *P. vivax* PCR-uncorrected 42-d ACPR. Non-inferiority and superiority designs were used for falciparum and vivax malaria, respectively. Because the artemisinin-naphthoquine regimen involved three doses rather than the manufacturer-specified single dose, the first 188 children underwent detailed safety monitoring. Of 2,542 febrile children screened, 267 were randomized, and 186 with falciparum and 47 with vivax malaria completed the 42-d follow-up. Both ACTs were safe and well tolerated. *P. falciparum* ACPRs were 97.8% and 100.0% in artemether-lumefantrine and artemisinin-naphthoquine-treated patients, respectively (difference 2.2% [95% CI −3.0% to 8.4%] versus −5.0% non-inferiority margin, *p = *0.24), and *P. vivax* ACPRs were 30.0% and 100.0%, respectively (difference 70.0% [95% CI 40.9%–87.2%], *p<*0.001). Limitations included the exclusion of 11% of randomized patients with sub-threshold parasitemias on confirmatory microscopy and direct observation of only morning artemether-lumefantrine dosing.

**Conclusions:**

Artemisinin-naphthoquine is non-inferior to artemether-lumefantrine in PNG children with falciparum malaria but has greater efficacy against vivax malaria, findings with implications in similar geo-epidemiologic settings within and beyond Oceania.

**Trial registration:**

Australian New Zealand Clinical Trials Registry ACTRN12610000913077

*Please see later in the article for the Editors' Summary*

## Abstract 2

In a randomized controlled trial Tim Davis and colleagues investigate Artemisinin-naphthoquine versus artemether-lumefantrine for the treatment of *P. falciparum* and *P. vivax* malaria.

*Please see later in the article for the Editors' Summary*

## Editors' Summary

**Background:**

Malaria is a mosquito-borne parasitic disease that kills more than 600,000 people (mainly young children in sub-Saharan Africa) every year. *Plasmodium falciparum* causes most of these deaths, but *P. vivax* is the most common and most widely distributed cause of malaria outside sub-Saharan Africa. Infection with malaria parasites causes recurring flu-like symptoms and must be treated promptly with antimalarial drugs to prevent the development of anemia and potentially fatal damage to the brain and other organs. In the past, malaria was treated with “monotherapies” such as chloroquine, but the parasites quickly developed resistance to many of these inexpensive drugs. The World Health Organization now recommends artemisinin combination therapy (ACT) for first-line treatment of malaria in all regions where there is drug-resistant malaria. In ACT, artemisinin derivatives (fast-acting antimalarial drugs that are rapidly cleared from the body) are used in combination with a slower acting, more slowly eliminated partner drug to prevent reemergence of the original infection and to reduce the chances of the malaria parasites becoming resistant to either drug.

**Why Was This Study Done?:**

Because falciparum and vivax malaria respond differently to antimalarial drugs, wherever there is transmission of both types of malaria but limited facilities for species-specific malaria diagnosis—as in Papua New Guinea—compromises have to be made about which ACT should be used for the treatment of malaria. Thus, Papua New Guinea's national guidelines recommend artemether-lumefantrine, which is effective against the more deadly *P. falciparum*, for first-line treatment of uncomplicated (mild) malaria even though this ACT is ineffective against the more common *P. vivax*. In this open-label randomized trial (a study in which participants are randomly assigned to receive different drugs but know which drug they are being given), the researchers ask whether an alternative ACT might be preferable for the treatment of uncomplicated malaria in young children in Papua New Guinea by comparing outcomes after treatment with artemether-lumefantrine versus artemisinin-naphthoquine (an ACT that should be more effective against vivax malaria than artemether-lumefantrine because naphthoquine stays in the body longer than lumefantrine). Specifically, the researchers test the non-inferiority of artemisinin-naphthoquine compared to artemether-lumefantrine for the treatment of falciparum malaria (whether artemisinin-naphthoquine is not worse than artemether-lumefantrine) and the superiority of artemisinin-naphthoquine compared to artemether-lumefantrine for the treatment of vivax malaria (whether artemisinin-naphthoquine is better than artemether-lumefantrine).

**What Did the Researchers Do and Find?:**

The researchers assigned nearly 250 children (aged 0.5 to 5 years) with falciparum malaria, vivax malaria, or both types of malaria to receive six doses of artemether-lumefantrine over three days or three daily doses of artemisinin-naphthoquine. They then followed the children to see how many children in each treatment group and with each type of malaria were free of malaria 42 days after treatment (an “adequate clinical and parasitological response”). Among the patients originally infected with *P. falciparum*, 97.8% of those treated with artemether-lumefantrine and 100% of those treated with artemisinin-naphthoquine were clear of their original *P. falciparum* infection (though some had acquired a new *P. falciparum* infection) 42 days after treatment. By contrast, among the patients infected with *P. vivax*, 30% of those treated with artemether-lumefantrine and 100% of those treated with artemisinin-naphthoquine were clear of *P. vivax* infection 42 days after treatment. Both ACTs were safe and well tolerated.

**What Do These Findings Mean?:**

These findings indicate that artemisinin-naphthoquine was non-inferior to artemether-lumefantrine for the treatment of uncomplicated falciparum malaria among young children in Papua New Guinea and had greater efficacy than artemether-lumefantrine against vivax malaria. The accuracy of these findings may be limited by several aspects of the study design. For example, not all the artemether-lumefantrine doses were directly observed, so some children may not have received the full treatment course. Moreover, because all the study participants lived in coastal communities in Papua New Guinea where malaria is highly endemic, treatment responses among children living in areas with lower levels of malaria transmission might be different. Nevertheless, these findings suggest that artemisinin-naphthoquine should be considered alongside other ACTs for the treatment of uncomplicated malaria in regions where there is transmission of multiple *Plasmodium* species and that artemisinin-naphthoquine may be better than artemether-lumefantrine for the treatment of uncomplicated malaria in young children in regions where *P. vivax* predominates.

**Additional Information:**

Please access these websites via the online version of this summary at http://dx.doi.org/10.1371/journal.pmed.1001773.

- Information is available from the World Health Organization on malaria (in several languages); the World Malaria Report 2013 provides details on the current global malaria situation, including information on malaria in Papua New Guinea; the World Health Organization's Guidelines for the Treatment of Malaria is available
- The US Centers for Disease Control and Prevention provides information on malaria (in English and Spanish), including personal stories about malaria
- Information is available from the Roll Back Malaria Partnership on the global control of malaria, including information about malaria in Papua New Guinea, malaria in children, and ACTs
- The Malaria Vaccine Initiative has a fact sheet on Plasmodium vivax malaria
- MedlinePlus provides links to additional information on malaria (in English and Spanish)
- More information about this trial is available

## Introduction

Malaria control programs incorporating artemisinin combination therapy (ACT) have contributed to a decline in malaria morbidity and mortality worldwide, renewing interest in global eradication [1]. However, in geo-epidemiologic settings outside sub-Saharan Africa where there is transmission of multiple *Plasmodium* species, *P. vivax* remains a major obstacle to eradication because of its complex life cycle and transmission biology [2]. There is the potential for late relapses from liver-stage hypnozoites, but also growing evidence that the burden of disease and the potential for death from acute *P. vivax* infections have both been underestimated [3],[4].

Transmission of both *P. falciparum* and *P. vivax* can complicate the choice of antimalarial treatment. A recent trial of ACTs involving children from Papua New Guinea (PNG) with uncomplicated malaria showed that the most efficacious were artemether-lumefantrine for *P. falciparum* and dihydroartemisinin-piperaquine for *P. vivax* infections [5]. Because of the impracticality of species-specific treatment where there are limited diagnostic facilities, and because falciparum malaria more often progresses to complications and death, artemether-lumefantrine was chosen as the first-line therapy under 2009 PNG national guidelines [6]. This recommendation means that potentially preventable morbidity and mortality due to vivax malaria remain of concern [7].

There is a need for assessment of other ACTs that might replicate the high cure rate of artemether-lumefantrine in falciparum malaria while ensuring that vivax malaria is also effectively treated in PNG and similar epidemiologic settings. Of the few available alternatives, artemisinin-naphthoquine has been marketed in a range of countries in Africa, Asia, and Oceania as single-dose treatment based on limited pharmacologic, efficacy, and safety data in adults [8],[9] and without World Health Organization (WHO) prequalification. Because of its commercial availability in PNG, and as a prelude to a formal safety and efficacy comparison with artemether-lumefantrine, we performed pharmacokinetic dose-finding studies in PNG children aged 5–10 y, which provided evidence that a 3-d daily artemisinin-naphthoquine regimen was well tolerated, safe, and efficacious for uncomplicated malaria [10],[11]. This regimen satisfies the WHO requirement that ACTs be administered over 3 d to retard parasite drug resistance and improve efficacy [12]. In addition, the elimination half-life of naphthoquine [10] is longer than that of piperaquine [13], suggesting that it should be more effective in suppressing *P. vivax* relapses in patients treated for vivax or falciparum malaria [5].

The aim of the present study was to compare the efficacy of three daily artemisinin-naphthoquine doses to that of a six-dose, 3-d artemether-lumefantrine regimen in PNG children aged 0.5–5 y with uncomplicated malaria. We hypothesized that artemisinin-naphthoquine would be well tolerated and safe in this younger vulnerable pediatric population, and that its efficacy would be (i) non-inferior to that of artemether-lumefantrine for falciparum malaria and (ii) superior to that of artemether-lumefantrine for vivax malaria.

## Methods

### Study Design, Setting, and Approvals

This open-label, parallel-group trial was conducted at the Mugil and Alexishafen Health Centers, Madang Province, PNG, from 28 March 2011 to 22 April 2013. The primary efficacy endpoints were (i) reappearance of *P. falciparum* within 42 d of treatment after correction for reinfections identified by polymerase chain reaction (PCR) genotyping of polymorphic parasite loci, and (ii) appearance of any *P. vivax* parasitemia within 42 d after treatment for vivax malaria. Secondary endpoints for falciparum malaria, assessed over 42 d, were (i) reappearance of PCR-uncorrected *P. falciparum* parasitemia, (ii) appearance of *P. vivax* parasitemia, and (iii) persistence/appearance of *P. falciparum* gametocytes. For vivax malaria, secondary endpoints were (i) persistence/appearance of *P. vivax* gametocytes over 42 d and (ii) reappearance of PCR-corrected *P. vivax* over 42 d (added post hoc when genotyping techniques became available [14]). Although the WHO recommendation is 28 d of post-treatment monitoring [15], monitoring can be extended to 42 d in the case of ACT partner drugs with a long half-life [12]. Analyses of key outcomes were thus performed at both 28 d and 42 d. Safety assessment included conventional clinical and laboratory monitoring as well as electrocardiographic indices.

Since artemether-lumefantrine has a high (>95%) day-42 adequate parasitologic and clinical response (ACPR) for falciparum malaria in the PNG pediatric population [5], a non-inferiority study design was selected. A sample size of 220 per falciparum malaria treatment arm was chosen based on the assumption that the *P. falciparum* day-42 PCR-corrected ACPR for artemether-lumefantrine would be 95.2% (as in our previous study [5]), a 5% non-inferiority margin for artemisinin-naphthoquine or artesunate-pyronaridine (approximating the 10% failure rate recommended by WHO for change in treatment policy [12]), 25% attrition, 5% type 1 error (one-tailed), and 80% power. For vivax malaria, given that artemether-lumefantrine has a low cure rate and that dihydroartemisinin-piperaquine was twice as efficacious as artemether-lumefantrine in PNG children in our previous study [5], a superiority study design was used. A sample size of 60 children per arm was selected under the assumption that the day-42 uncorrected *P. vivax* ACPR for artemisinin-naphthoquine or artesunate-pyronaridine would be superior (at least double the 30.3% ACPR for artemether-lumefantrine [5]), with 25% attrition, 5% type 1 error (two-tailed), and 90% power.

The original study design involved a comparison of conventional artemether-lumefantrine with both artemisinin-naphthoquine and artesunate-pyronaridine. Artesunate-pyronaridine was not initially available because concerns regarding hepatotoxicity were being addressed [16]. The subsequent recommendation that this ACT be limited to a single lifetime dose made its use untenable for malaria treatment programs in countries such as PNG where children are at risk of repeated episodes of malaria [17], and, as a result, the trial was conducted as a two-arm comparison. A prespecified artemisinin-naphthoquine safety assessment was scheduled after 50 patients, as well as further interim efficacy analyses and safety assessments that were scheduled primarily to determine whether artemisinin-naphthoquine violated the non-inferiority margin in the case of falciparum malaria or unexpected toxicity had emerged.

The study was approved by the PNG Institute of Medical Research Review Board, the Medical Research Advisory Committee of PNG, and the University of Western Australia Human Research Ethics Committee. Written informed consent was obtained from parents/guardians of all children.

### Patients

Children aged 0.5–5 y presenting with an axillary temperature >37.5°C or a history of fever during the previous 24 h were screened using on-site blood film microscopy. Those with *P. falciparum* (>1,000 asexual parasites/µl whole blood) or *P. vivax* (>250/µl) were eligible if (i) there were no features of severity [18], (ii) they had not taken a study drug in the previous 14 d, (iii) there was no history of allergy to study drugs, and (iv) there was no evidence of another infection or co-morbidity.

### Clinical Procedures

An initial standardized clinical assessment was performed, and blood was drawn for measurement of hemoglobin and blood glucose (Hemocue 201+, Hemocue, Ängelholm, Sweden), as well as hepatic and renal function (Vitros DT60 II system, Ortho Clinical Diagnostics, Mulgrave, Victoria, Australia) and a full blood count (Coulter Ac·T diff, Beckman Coulter, Brea, California, US). A 12-lead electrocardiograph was taken for measurement of the QT interval. Each QT interval was corrected for heart rate using Bazett's formula (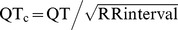) to allow comparisons with other studies in the PNG pediatric population involving antimalarial drugs with effects on ventricular repolarization in which this correction has been applied [11],[19],[20].

Based on computer-generated block randomization (24 children per site), eligible patients were allocated 1∶1 to artemether-lumefantrine (1.7 mg/kg artemether plus 10 mg/kg lumefantrine; Novartis Pharma, Basel, Switzerland) twice daily for 3 d or to artemisinin-naphthoquine (20 mg/kg artemisinin plus 8 mg/kg naphthoquine; Kunming Pharmaceutical Corporation, Yunnan, China) daily for 3 d. Randomization was independent of *Plasmodium* species. Allocated treatments were concealed in sealed numbered envelopes that were opened in sequence by study medical or nursing staff, and the specified treatment was administered. As recommended by the respective manufacturers, artemether-lumefantrine was administered as 1–3 whole tablets per dose with 250 ml of milk to optimize bioavailability [21], and artemisinin-naphthoquine as 1–4 whole tablets per dose with water.

Treatments were not blinded, primarily because the endpoints were based on objective clinical and parasitologic criteria. In addition, we sought to simplify drug administration as much as possible to maximize patient adherence and retention, including only once daily dosing and the avoidance of potential gastrointestinal side effects with the unnecessary ingestion of milk in children allocated artemisinin-naphthoquine [10]. Only the evening artemether-lumefantrine dose was unsupervised, being administered at home by parents/guardians who were instructed as to the importance of dosing with milk, which was supplied for this purpose. Adherence was verified by study staff the following morning in each case, and a day-7 plasma lumefantrine concentration was determined for each child. Children vomiting within 30 min of drug administration were to be retreated.

Standardized assessment, including axillary temperature and blood film microscopy, was repeated on days 1, 2, 3, 7, 14, 28, and 42. Blood was drawn for full blood count and hepatorenal function, and an electrocardiograph was taken, on days 0, 3, and 7. An additional electrocardiograph was performed 4 h after the day-2 dose (at the predicted peak plasma naphthoquine concentration [10]) in artemisinin-naphthoquine-treated children and in a convenience sample of 30 children at the same time after the morning dose of artemether-lumefantrine. All blood films were reexamined independently by two skilled microscopists who were blind to allocated treatment. Parasite density was calculated from the number of parasites per 200–500 leucocytes and an assumed leucocyte count of 8,000/µl. Slides discrepant for positivity/negativity, speciation, or density (>3× difference) were adjudicated by a senior microscopist.

Efficacy was assessed using WHO definitions [15]. Early treatment failure (ETF) was defined as the development of signs of severity or an inadequate parasitologic response by day 3. Any child developing parasitemia between days 4 and 42 was considered a late parasitologic failure (LPF) or, if febrile, late clinical failure (LCF). An ACPR was recorded otherwise. *P. falciparum* reinfection and recrudescence were distinguished using PCR genotyping of merozoite surface protein 2 (MSP2) and MSP1 based on paired samples collected on day 0 and at reappearance [22],[23]. *P. vivax* recrudescence was determined based on genotyping of MSP1F3 and Microsatellite 16 [14]. Parasite clearance time (PCT) and fever clearance time (FCT) were defined as the times to the first of two consecutive assessments at which the child was afebrile and slide-negative, respectively.

Day-7 plasma lumefantrine concentrations were determined using a validated ultra-high-performance liquid chromatography–tandem mass spectrometry assay as previously described [24],[25]. The inter-day and intra-day variability were ≤11.2% and ≤9.6%, respectively, at concentrations over the linear range between 20 and 20,000 ng/ml.

### Statistical Analysis

Per-protocol prespecified analyses included children with complete follow-up or a confirmed treatment failure, and excluded those treated for malaria without confirmatory microscopy, those for whom the alternative *Plasmodium* species was detected, and those who defaulted from follow-up despite repeated attempts at contact. These excluded patients were retained in prespecified modified intention-to-treat analyses utilizing (i) a worst-case approach (ETF assumed for day-3 exclusions, LPF/LCF otherwise) and (ii) a best-case approach (all missing follow-up blood films assumed parasite-negative) [5].

Kaplan-Meier estimates were computed for each endpoint defined by parasite species. Statistical analysis was performed using IBM SPSS Statistics version 20 (IBM Corporation, Somers, New York, US) and STATA/IC 11.2 (StataCorp, College Station, Texas, US). Two-sample comparisons for normally distributed variables were by Student's *t* test, for non-normally distributed variables by Mann-Whitney *U* test, and for proportions by Fisher's exact test. Safety and tolerability were assessed from the incidence of symptoms/signs to day 7 using Poisson regression. Kaplan-Meier estimates were computed for each endpoint by *Plasmodium* species, and treatments compared by log-rank test. Differences in parasitemia, gametocytemia, and hemoglobin concentration by treatment type and time were assessed using generalized linear modeling with Bonferroni correction to adjust for multiple pairwise comparisons, and with adjustment for parasitemia in the case of hemoglobin. Unless otherwise stated, all *p*-values are two-tailed, with *p*<0.05 taken as significant.

## Results

### Trial Profile

**Figure 1:**
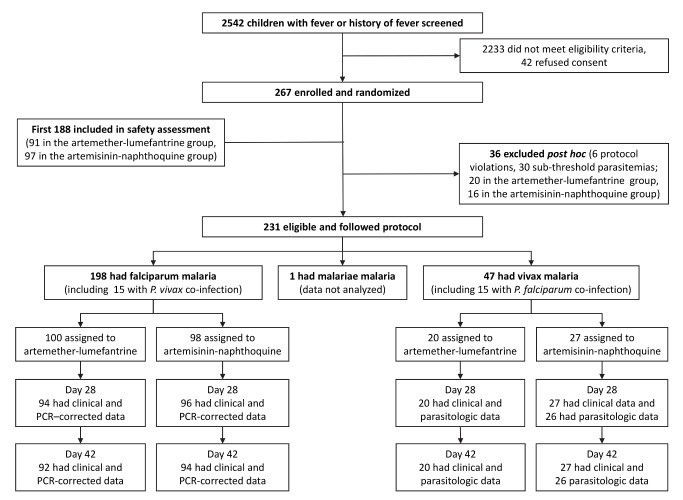
Trial profile showing numbers of patients remaining in the study from screening to day-42 assessment. PCR-corrected denotes correction for reinfections identified by polymerase chain reaction genotyping of polymorphic parasite loci.

The first patient was recruited on 28 March 2011, and the last follow-up was completed on 22 April 2013. No adverse events occurred in the first 50 patients. A further safety assessment was scheduled when one-third of the total planned sample size of 560 had been recruited. Safety data were available for 91 artemether-lumefantrine-treated and 97 artemisinin-naphthoquine-treated children at that time (including those excluded post hoc; see Figure 1). No significant drug-related adverse events had occurred. Detailed safety monitoring was therefore stopped, but scheduled visits still included basic clinical/laboratory parameters including axillary temperature, blood film microscopy, and hemoglobin concentration. A prespecified interim efficacy analysis was performed when nearly 50% of the sample had been recruited (267 of 560). This analysis showed that artemisinin-naphthoquine was non-inferior for falciparum malaria and superior for vivax malaria compared with artemether-lumefantrine (see below), justifying cessation of the trial.

Of 267 randomized children, 36 (13.5%) were excluded post hoc because of protocol violations (20 allocated to artemether-lumefantrine and 16 to artemisinin-naphthoquine; see Figure 1). Six children had not taken study treatment as stipulated (three allocated to each treatment), seven were slide-negative by expert microscopy (four allocated to artemether-lumefantrine and three to artemisinin-naphthoquine), and 23 had sub-threshold parasite densities by expert microscopy (13 allocated to artemether-lumefantrine and ten to artemisinin-naphthoquine). There were no significant differences between treatment allocations for the proportions of total exclusions or individual reasons for exclusion (*p>*0.47 in each case). In addition, there were no differences between the treatments allocated to the 23 excluded patients who had falciparum malaria or between the treatments allocated to the six with vivax malaria (*p>*0.67). The 15 children (6.5% of the randomized sample) presenting with mixed *P. falciparum*/*P. vivax* infections at densities above each species-specific threshold were included in both species arms [5]. A single patient with *P. malariae* infection completed all study procedures but was not included in analyses.

Of the remaining 230 children, the day-42 retention was ≥92% for all species/treatment groups. Ten children (six allocated to artemether-lumefantrine and four to artemisinin-naphthoquine, all with falciparum malaria) withdrew during follow-up. None of these children had experienced adverse effects; they had all relocated outside the study catchment areas. Two children with *P. falciparum* infections in the artemether-lumefantrine arm were found to have symptomatic vivax malaria on day 28 with parasitemias of 21,137/µl and 1,609/µl, respectively. These patients were retreated with artemether-lumefantrine under national guidelines [6] at that time and were excluded from the per-protocol analysis. The remaining children with *P. vivax* detected during follow-up after treatment for falciparum malaria were mostly asymptomatic and had lower parasitemias, and were either not treated or given artemether-lumefantrine on day 42 after assessment. One child with *P. vivax* at recruitment who was allocated to artemisinin-naphthoquine attended all follow-up visits but did not have parasitologic data at day 28 and was excluded from the per-protocol analysis. All children allocated artemether-lumefantrine had detectable lumefantrine in plasma on day 7, with a median concentration (192 ug/l [interquartile range 122–352]) consistent with all six doses having been administered successfully [24],[25].

### Safety Assessment

There were no significant differences in baseline demographic and anthropometric characteristics (age, sex, weight, height, and mid-upper-arm circumference), vital signs (axillary temperature, heart rate, and respiratory rate), hematologic parameters (white and red blood cell counts, platelet count, and hemoglobin concentration), or blood glucose concentrations between the 91 children allocated to artemether-lumefantrine and the 97 who received artemisinin-naphthoquine in the detailed safety assessment (*p*≥0.21; see Table 1). No child in either group had a baseline serum creatinine concentration more than three times the upper limit of the reference range (ULRR) for PNG children [26] (≥112 µmol/l), but two children in the artemether-lumefantrine group had a baseline serum alanine aminotransferase (ALT) concentration more than three times the ULRR (≥130 U/l; see Table 2). Consistent with malaria-associated hemolysis, approximately one-third of the children in both groups had a baseline serum total bilirubin concentration more than three times the ULRR (≥24 µmol/l), but there was no between-group difference in this proportion (*p = *0.60) and no children were considered to have jaundice.

**Table 1:** Demographic, clinical, and laboratory parameters as part of safety monitoring.

Parameter	Artemether-Lumefantrine		Artemisinin-Naphthoquine		*p*-Value
	*n*	Mean ± SD, Median (IQR), or Percentage	*n*	Mean ± SD, Median (IQR), or Percentage	
**Day 0**					
Age (months)	91	44.2±14.2	97	42.7±4.6	0.50
Sex (percent male)	91	44.0%	97	49.5%	0.47
Body weight (kg)	90	12.7±2.1	95	12.7±2.5	0.87
Height (cm)	83	93.8±10.3	91	92.9±10.3	0.55
Mid-upper-arm circumference (cm)	90	15.0 (14.0–15.0)	94	14.5 (14.0–15.5)	0.39
Axillary temperature (°C)	90	38.0±1.3	95	38.1±1.4	0.74
Pulse rate (/min)	89	123±20	92	126±21	0.26
Respiratory rate (/min)	89	32 (28–37)	93	32 (26–38)	0.55
White blood cells (× 10^9^/l)	85	6.9 (5.4–9.8)	91	7.4 (5.0–9.3)	0.78
Red blood cells (× 10^12^/l)	85	3.68 (3.03–4.27)	91	3.77 (3.23–4.34)	0.55
Platelets (× 10^9^/l)	84	85 (64–132)	91	102 (63–137)	0.78
Hemoglobin (g/l)	89	93 (81–104)	94	91 (81–102)	0.31
Blood glucose (mmol/l)	83	6.4 (5.7–7.6)	87	6.7 (5.8–7.4)	0.59
Reticulocytes (percent)	48	1.2% (0.6%–2.9%)	48	0.8% (0.6%–2.0%)	0.21
**Day 3**					
Axillary temperature (°C)	88	36.4±0.4	94	36.2±0.5	0.002
Pulse rate (/min)	87	107±15	94	107±17	0.36
Respiratory rate (/min)	88	28 (24–32)	94	28 (24–32)	0.95
White blood cells (× 10^9^/l)	87	7.0 (5.5–8.7)	90	7.1 (5.9–8.5)	0.71
Red blood cells (× 10^12^/l)	87	3.47 (3.03–3.91)	90	3.57 (3.03–4.09)	0.65
Platelets (× 10^9^/l)	87	142 (107–180)	90	149 (102–188)	0.63
Hemoglobin (g/l)	88	87 (75–96)	94	86 (73–99)	0.92
Blood glucose (mmol/l)	78	5.9 (5.3–6.5)	81	5.8 (5.3–6.6)	0.73
Reticulocytes (percent)	49	1.2% (0.6%–3.4%)	49	0.8% (0.5%–1.3%)	0.10
**Day 7**					
Axillary temperature (°C)	89	36.5±0.6	92	36.6±0.6	0.51
Pulse rate (/min)	89	105±18	94	106±19	0.68
Respiratory rate (/min)	89	28 (24–31)	94	28 (24–32)	0.41
White blood cells (× 10^9^/l)	87	8.1 (6.1–9.5)	88	8.4 (7.1–10.7)	0.01
Red blood cells (× 10^12^/l)	87	3.64 (3.34–4.25)	88	3.61 (3.15–4.19)	0.43
Platelets (× 10^9^/l)	87	245 (191–337)	88	257 (187–315)	0.59
Hemoglobin (g/l)	87	93 (80–102)	94	91 (80–101)	0.64
Blood glucose (mmol/l)	80	5.9 (5.3–6.5)	82	6.1 (5.4–6.5)	0.70
Reticulocytes (percent)	49	3.6% (1.4%–6.2%)	49	2.3% (1.3%–3.6%)	0.05

IQR, interquartile range; SD, standard deviation.

**Table 2:** Biochemical measurements in the safety sub-study by allocated treatment.

Measurement	Artemether-Lumefantrine	Artemisinin-Naphthoquine	*p*-Value
**Day 0 (baseline)**			
Serum creatinine (µmol/l)	38 (29–47) (*n = *72, 79%)	23 (28–43) (*n = *73, 75%)	0.09
Elevated creatinine*, *n* (percent)	0 (0%)	0 (0%)	>0.99
Serum ALT (U/l)	20 (13–35) (*n = *72, 79%)	20 (13–28) (*n = *67, 69%)	0.86
Elevated ALT*, *n* (percent)	2 (2.8%)	0 (0%)	0.50
Serum total bilirubin (µmol/l)	20 (12–25) (*n = *74, 81%)	21 (12–26) (*n = *75, 77%)	0.77
Elevated bilirubin*, *n* (percent)	22 (29.7%)	26 (34.7%)	0.60
**Day 3**			
Serum creatinine (µmol/l)	36 (27–41) (*n = *74, 81%)	37 (31–44) (*n = *73, 75%)	0.23
Elevated creatinine*, *n* (percent)	0 (0%)	0 (0%)	>0.99
Serum ALT (U/l)	18 (16–20) (*n = *73, 80%)	20 (17–26) (*n = *67, 69%)	0.16
Elevated ALT*, *n* (percent)	1 (1.4%)	1 (1.5%)	>0.99
Serum total bilirubin (µmol/l)	14 (6–17) (*n = *70, 77%)	13 (6–17) (*n = *67, 69%)	0.94
Elevated bilirubin*, *n* (percent)	2 (2.9%)	1 (1.5%)	>0.99
**Day 7**			
Serum creatinine (µmol/l)	29 (22–34) (*n = *71, 78%)	28 (24–40) (*n = *71, 73%)	0.43
Elevated creatinine*, *n* (percent)	0 (0%)	0 (0%)	>0.99
Serum ALT (U/l)	18 (12–25) (*n = *70, 77%)	21 (16–29) (*n = *67, 69%)	0.03
Elevated ALT*, *n* (percent)	0 (0%)	0 (0%)	>0.99
Serum total bilirubin (µmol/l)	11 (8–16) (*n = *71, 78%)	14 (9–15) (*n = *69, 71%)	0.63
Elevated bilirubin*, *n* (percent)	2 (2.8%)	5 (7.2%)	0.27

Data are median (interquartile range) or *n* (percent).

*Elevated serum creatinine (≥112 µmol/l), serum ALT (≥130 U/l), and total serum bilirubin (≥23.8 µmol/l) are defined as>3 times the upper limit of normal values in PNG children aged <5 y [26].

Both treatments were well tolerated, and there were no between-group differences in the incidence rates of reported/observed signs and symptoms during the first 7 d of follow-up, apart from a significantly greater incidence of abdominal pain in the artemisinin-naphthoquine-treated patients (incidence rate 11.6% versus 7.8% in the artemether-lumefantrine group; incidence rate ratio 1.48 [95% CI 1.03–2.13], *p = *0.033). This symptom was mild and short-lived in all cases (see Table 3). On day 3, one child in each group had a serum ALT more than three times the ULRR, but both were afebrile and asymptomatic, and this abnormality had resolved in both cases by day 7 (see Table 2). Most children had a normal serum bilirubin by day 7. No child became hypoglycemic. Other statistically significant between-group differences in vital signs and hematologic parameters on days 3 and 7 were not clinically significant.

**Table 3:** Incidence rate of main reported or observed signs and symptoms during the first 7 d of follow-up in randomized children, expressed as reports per 100 observations.

Sign/Symptom	Incident Rate (95% CI)		IRR (95% CI)	*p*-Value
	Artemether-Lumefantrine (*n = *130)	Artemisinin-Naphthoquine (*n = *127)		
Fever	27.9 (24.4–31.5)	30.7 (27.1–34.5)	1.10 (0.90–1.35)	0.37
Chills	5.8 (4.1–7.9)	6.3 (4.5–8.5)	1.08 (0.69–1.70)	0.72
Headaches	7.5 (5.6–9.8)	10.1 (7.9–12.8)	1.35 (0.93–1.97)	0.12
Child irritable/frequent crying	2.7 (1.6–4.2)	3.4 (2.1–5.11)	1.27 (0.67–2.41)	0.46
Trouble sleeping	0.9 (0.4–2.0)	1.3 (0.6–2.5)	1.37 (0.48–3.95)	0.56
Problems eating/breast feeding	12.4 (10.0–15.2)	11.7 (9.3–14.5)	0.94 (0.68–1.29)	0.70
Vomiting	4.2 (2.8–6.1)	5.8 (4.1–7.9)	1.37 (0.83–2.26)	0.21
Diarrhea	4.9 (3.3–6.8)	3.5 (3.2–5.3)	0.73 (0.42–1.26)	0.26
Abdominal pain	7.8 (5.8–10.1)	11.6 (9.2–14.3)	1.48 (1.03–2.13)	0.03
Cough	24.0 (20.8–27.5)	29.1 (25.6–32.8)	1.21 (0.98–1.50)	0.08
Difficulty breathing	0.9 (0.3–2.0)	0.3 (0.0–1.2)	0.34 (0.07–1.70)	0.19
Rhinorrhea	23.9 (20.6–27.4)	22.1 (18.9–25.6)	0.93 (0.74–1.17)	0.53
Skin rash (reported)	3.3 (2.0–5.0)	3.1 (1.9–4.7)	0.93 (0.50–1.73)	0.82
Skin rash (observed)	0.3 (0.0–1.1)	1.1 (0.5–2.3)	3.60 (0.75–17.33)	0.11
Skin lesion	2.7 (1.6–4.2)	2.4 (1.4–4.0)	0.91 (0.45–1.82)	0.79
Pallor	6.7 (4.9–8.9)	9.3 (7.2–11.9)	1.39 (0.94–2.06)	0.10
Crackles at lung bases	3.0 (1.8–4.6)	1.6 (0.8–2.9)	0.54 (0.25–1.16)	0.12
Presence of enlarged spleen	22.9 (19.7–26.4)	26.5 (23.1–30.2)	1.16 (0.92–1.44)	0.20

Poisson regression with follow-up time as the exposure was used to derive incident rate ratios with artemether-lumefantrine as reference. Data on signs/symptoms from ten children were lost prior to database entry.

IRR, incident rate ratio.

**Figure 2:**
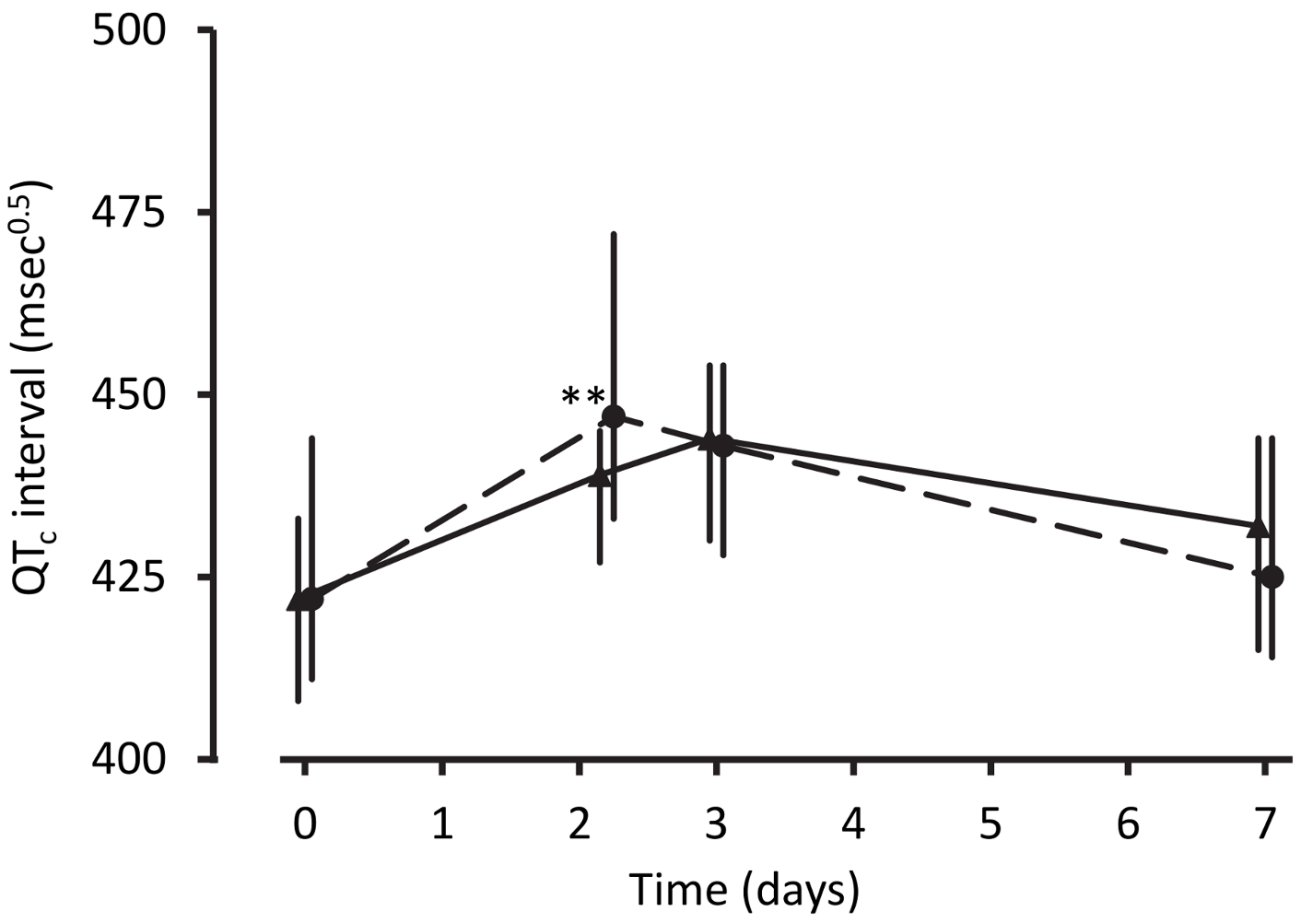
Electrocardiographic QT_c_ intervals after treatment for uncomplicated malaria. Data are median and interquartile range (vertical bars) for artemether-lumefantrine (▴, solid lines) and artemisinin-naphthoquine (•, dashed lines). **p<0.01 for between-treatment comparisons.

Despite the technical challenges associated with performing electrocardiography in unwell children in a rural clinic, interpretable traces were obtained from between 68% and 88% of the children in each group at each time point. The QT_c_ increased from baseline to 4 h after the last dose of artemisinin-naphthoquine by a median of 28 msec^0.5^ (interquartile range 18–36) (*p<*0.01 versus artemether-lumefantrine-treated children; see Figure 2 and Table 4). Most values had returned to baseline by day 7, with a prolongation relative to baseline of 5 msec^0.5^ (interquartile range 2–9). There were no significant changes in QT_c_ in the artemether-lumefantrine group. Twenty-six of 102 children (25.5%, all but one in the much larger artemisinin-naphthoquine group) with an interpretable electrocardiograph on day 2 had QT_c_>460 msec^0.5^. The longest QT_c_ at this time point was 505 msec^0.5^, in an artemisinin-naphthoquine-treated child. No dysrhythmias, dyspnea, syncope, or other transient neurologic events were recorded during follow-up.

**Table 4:** Electrocardiographic QT_c_ measurements by allocated treatment.

QT_c_ (msec^0.5^)	Artemether-Lumefantrine	Artemisinin-Naphthoquine	*p*-Value
**Day 0**	*n = *68	*n = *80	
Median (IQR)	422 (408–443)	422 (411–444)	0.96
≤440	73.5%	70.0%	0.25
441–450	11.8%	17.5%	
451–460	10.3%	3.8%	
>460	4.4%	8.8%	
**Day 2**	*n = *27	*n = *75	
Median (IQR)	439 (427–445)	447 (433–472)	0.003
≤440	55.6%	34.7%	0.005
441–450	33.3%	20.0%	
451–460	7.4%	12.0%	
>460	3.7%	33.3%	
**Day 3**	*n = *62	*n = *84	
Median (IQR)	444 (430–454)	443 (428–454)	0.82
≤440	41.9%	44.0%	0.87
441–450	30.6%	27.4%	
451–460	14.5%	11.9%	
>460	12.9%	16.7%	
**Day 7**	*n = *69	*n = *85	
Median (IQR)	432 (415–444)	425 (414–444)	0.64
≤440	66.7%	67.1%	0.83
441–450	17.4%	21.2%	
451–460	11.6%	8.2%	
>460	4.3%	3.5%	

IQR, interquartile range.

The only severe adverse event was considered non-drug related. A 48-mo-old child allocated to artemisinin-naphthoquine was hospitalized with, and treated successfully for, lobar pneumonia diagnosed on day 23 of follow-up. This patient was negative by both microscopy and PCR for malaria at the time of re-presentation.

### Antimalarial Efficacy

The two falciparum malaria treatment groups were well matched for age, sex, anthropometric measures, vital signs, and other characteristics (see Table 5). Initial fever and parasite clearance were prompt. Most children in each group (≥88.0%) were afebrile, and approximately one-third were parasite negative, by day 1, and almost all had cleared their fever (100.0% in the artemether-lumefantrine arm versus 98.0% in the artemisinin-naphthoquine arm, *p = *0.25) and parasitemia (99.0% versus 98.0%, *p>*0.99) by day 3 (see Table 6).

**Table 5:** Baseline characteristics of patients classified by *Plasmodium* species and allocated treatment.

Characteristic	Falciparum Malaria			Vivax Malaria		
	Artemether-Lumefantrine (*n = *100)	Artemisinin-Naphthoquine (*n = *98)	*p*-Value	Artemether-Lumefantrine (*n = *20)	Artemisinin-Naphthoquine (*n = *27)	*p*-Value
**Male sex (percent)**	50.0%	57.1%	0.32	40.0%	40.7%	>0.99
**Age (months)**	43.4±15.6	44.8±15.7	0.54	37.1±15.2	36.0±13.9	0.81
**Weight (kg)**	12.6±2.4	12.8±2.6	0.58	12.2±2.2	11.9±2.2	0.63
**Mid-upper-arm circumference (cm)**	14.4±1.1	14.5±1.3	0.62	14.1±0.9	14.4±0.9	0.26
**Body mass index (kg/m^2^)**	14.4±1.7	14.7±2.0	0.38	14.5±1.3	14.8±1.8	0.56
**Heart rate (/min)**	123±21	125±22	0.50	121±24	120±18	0.93
**Respiratory rate (/min)**	32±8	32±9	0.86	34±11	33±13	0.79
**Axillary temperature (°C)**	38.2±1.3	38.2±1.4	0.82	38.2±1.5	37.9±1.7	0.52
**Enlarged spleen (percent)**	49.4%	53.4%	0.65	52.9%	33.3%	0.34
**Parasite density (/µl whole blood)**	23,277 (9,447–45,613)	24,976 (13,674–51,867)	0.33	6,146 (1,545–18,893)	7,252 (1,372–15,724)	0.86
**Hemoglobin (g/l)**	92.0±17.0	90.9±16.9	0.66	92.6±17.9	98.0±16.3	0.29
**Blood glucose (mmol/l)**	6.9±2.1	6.7±1.9	0.46	6.2±2.4	7.1±1.8	0.21
**Total drug dose over 3 d (mg/kg)**						
Artemether	12.0 [8.6–20.0]			11.2 [8.6–16.0]		
Lumefantrine	72.0 [51.4–120.0]			67.4 [51.4–96.0]		
Artemisinin		62.5 [37.5–75.0]			68.2 [48.7–75.0]	
Naphthoquine		25.0 [15–30.0]			27.3 [19.5–30.0]	

Data are percentage, mean ± standard deviation, median (interquartile range), or median [absolute range].

**Table 6:** Fever and parasite clearance times by *Plasmodium* species and allocated treatment.

Response Measure	*P. falciparum*			*P. vivax*		
	Artemether-Lumefantrine (*n = * 100)	Artemisinin-Naphthoquine (*n = * 98)	*p*-Value	Artemether-Lumefantrine (*n = * 20)	Artemisinin-Naphthoquine (*n = * 27)	*p*-Value
**Fever clearance (percent)**						
Day 1	88/100 (88.0%)	88/98 (89.8%)	0.82	17/20 (85.0%)	26/27 (96.3%)	0.30
Day 2	95/98 (96.9%)	93/98 (94.9%)	0.72	17/19 (89.5%)	26/27 (96.3%)	0.56
Day 3	99/99 (100%)	96/98 (98.0%)	0.25	19/19 (100%)	26/26 (100%)	—
Day 7	93/99 (93.9%)	94/98 (95.9%)	0.75	20/20 (100%)	24/26 (92.3%)	0.50
**Mean (95% CI) FCT (days)**	1.2 (1.1–1.3)	1.1 (1.0–1.2)	0.28	1.4 (1.0–1.8)	1.1 (0.9–1.4)	0.50
**Parasite clearance (percent)**						
Day 1	33/99 (33.3%)	30/98 (30.6%)	0.76	16/19 (84.2%)	23/27 (85.2%)	>0.99
Day 2	88/99 (88.9%)	86/98 (87.8%)	0.83	20/20 (100%)	27/27 (100%)	—
Day 3	97/98 (99.0%)	96/98 (98.0%)	>0.99	20/20 (100%)	27/27 (100%)	—
Day 7	98/98 (100%)	98/98 (100%)	—	20/20 (100%)	27/27 (100%)	—
**Mean (95% CI) PCT (days)**	1.8 (1.7–1.9)	1.8 (1.7–1.9)	0.66	1.2 (1.0–1.3)	1.1 (1.0–1.3)	0.99

Data are number/total (percentage) or mean (95% confidence interval).

**Figure 3:**
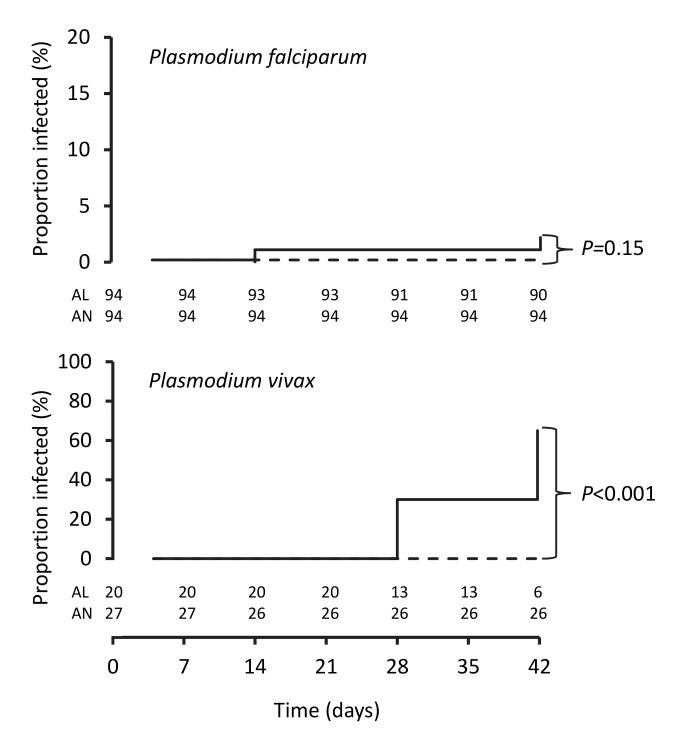
Kaplan-Meier plots showing percentages of patients positive for PCR-corrected *P. falciparum* and for PCR-uncorrected *P. vivax* after treatment. Data are for artemether-lumefantrine (solid lines) and artemisinin-naphthoquine (dashed lines). Numbers of children at risk at each time point are shown for artemether-lumefantrine (AL) and artemisinin-naphthoquine (AN). *p*-Values are for log-rank tests.

The PCR-corrected *P. falciparum* ACPR was 98.9% (95% CI 94.3%–100%; 93 of 94 children) and 100% (95% CI 95.2%–100%; 96 of 96 children) in the artemether-lumefantrine and artemisinin-naphthoquine groups, respectively, on day 28 (a secondary endpoint), and 97.8% (95% CI 91.6%–99.6%; 90 of 92 children) and 100% (95% CI 95.1%–100%; 94 of 94 children), respectively, on day 42 (a primary endpoint, *p = *0.15 by log-rank test; see Figure 3 and Table 7). Using these latter data and the same 5.0% non-inferiority margin, type 1 error probability, and power as in the original sample size estimates, the 186 children who completed follow-up exceeded the total of 52 required to show non-inferiority of artemisinin-naphthoquine for this primary endpoint [27]. In addition, the non-inferiority margin of −5.0% fell below the confidence interval for the difference between treatments (2.2% [95% CI −3.0% to 8.4%]), further evidence of non-inferiority.

**Table 7:** Per-protocol analysis of treatment responses in children with falciparum (after PCR correction) or vivax (PCR-uncorrected) malaria.

Endpoint	Number and Category	Total	Artemether-Lumefantrine	Artemisinin-Naphthoquine	Difference (95% CI)	*p*-Value
***P. falciparum,*** ** day 28**	*n*	190	94	96		
	ACPR *n*	189	93	96		
	Percent (95% CI)	99.5 (96.7–100)	98.9 (93.4–100)	100 (95.2–100)	1.1 (−3.8 to 6.6)	0.50 (one-sided)
	ETF/LCF/LPF (percent)	0/0/0.5	0/0/1.1	0/0/0		
***P. falciparum,*** ** day 42**	*n*	186	92	94		
	ACPR *n*	184	90	94		
	Percent (95% CI)	98.9 (95.8–99.8)	97.8 (91.6–99.6)	100 (95.1–100)	2.2 (−3.0 to 8.4)	0.24 (one-sided)
	ETF/LCF/LPF (percent)	0/0.5/0.5	0/1.1/1.1	0/0/0		
***P. vivax,*** ** day 28**	*n*	46	20	26		
	ACPR *n*	39	13	26		
	Percent (95% CI)	84.8 (70.5–93.2)	65.0 (41.0–83.7)	100 (84.0–100)	35.0 (10.7 to 59.1)	0.001 (two-sided)
	ETF/LCF/LPF (percent)	0/0/15.2	0/0/35.0	0/0/0		
***P. vivax,*** ** day 42**	*n*	46	20	26		
	ACPR *n*	32	6	26		
	Percent (95% CI)	69.6 (54.1–81.8)	30.0 (12.8–54.3)	100 (84.5–100)	70.0 (40.9 to 87.2)	<0.001 (two-sided)
	ETF/LCF/LPF (percent)	0/2.2/28.3	0/5.0/65.0	0/0/0		
***P. vivax*** ** after treatment for falciparum malaria, day 42**	*n*	188	94	94		
	ACPR *n*	156	62	94		
	Percent (95% CI)	83.0 (76.7–87.9)	66.0 (55.4–75.2)	100 (95.1–100)	34.0 (23.6 to 44.6)	<0.001 (one-sided)
	ETF/LCF/LPF (percent)	0/0/17.0	0/0/34.0	0/0/0		

The prespecified secondary endpoint of day-42 uncorrected ACPR was significantly greater in the artemisinin-naphthoquine group (100% [95% CI 95.1%–100%] versus 94.6% [95% CI 87.2%–98.0%] in the artemether-lumefantrine-treated children, *p = *0.028; see Table 8). In addition, the appearance of *P. vivax* after treatment for *P. falciparum*, also a secondary endpoint, was significantly less frequent in the artemisinin-naphthoquine group (0% versus 34.0% in in the artemether-lumefantrine-treated children, *p<*0.001; see Table 7). The modified intention-to-treat analyses showed results that were consistent with these findings (see Tables 9 and 10).

**Table 8:** Per-protocol secondary endpoint analysis of response in children with PCR-uncorrected *P. falciparum* reinfections or PCR-corrected *P. vivax* recrudescence by treatment allocation.

Endpoint	Number and Category	Total	Artemether-Lumefantrine	Artemisinin-Naphthoquine	Difference (95% CI)	*p*-Value
***P. falciparum,*** ** day 28**	*n*	190	94	96		
	ACPR *n*	188	92	96		
	Percent (95% CI)	98.9 (95.9–99.8)	97.9 (91.8–99.6)	100 (95.2–100)	2.1 (–3.0 to 8.2)	0.24
	ETF/LCF/LPF (percent)	0/0.5/0.5	0/1.1/1.1	0/0/0		
***P. falciparum,*** ** day 42**	*n*	186	92	94		
	ACPR *n*	181	87	94		
	Percent (95% CI)	97.3 (93.5– 99.0)	94.6 (87.2–98.0)	100 (95.1–100)	5.4 (−0.5 to 12.8)	0.028
	ETF/LCF/LPF (percent)	0/0.5/2.2	0/1.1/4.3	0/0/0		
***P. vivax,*** ** day 28**	*n*	46	20	26		
	ACPR *n*	42	16	26		
	Percent (95% CI)	91.3 (78.3–97.2)	80.0 (55.7–93.4)	100 (84.0–100)	20.0 (−0.9 to 44.3)	0.030
	ETF/LCF/LPF (percent)	0/2.2/6.5	0/5.0/15.0	0/0/0		
***P. vivax,*** ** day 42**	*n*	46	20	26		
	ACPR *n*	38	12	26		
	Percent (95% CI)	82.6 (68.1–91.7)	60.0 (36.4–80.0)	100 (84.0–100)	40.0 (14.4 to 63.6)	<0.001
	ETF/LCF/LPF (percent)	0/2.2/15.2	0/5.0/35.0	0/0/0		

**Table 9:** Modified intention-to-treat analysis of treatment response by treatment allocation utilizing a worst-case scenario (i.e., all instances of missing data during follow-up scored as treatment failure).

Endpoint	Number and Category	Total	Artemether-Lumefantrine	Artemisinin-Naphthoquine	Difference (95% CI)	*p*-Value
***P. falciparum,*** ** day 28**	*n*	198	100	98		
	ACPR *n*	189	93	96		
	Percent (95% CI)	95.5 (91.3–97.8)	93.0 (85.6–96.9)	98.0 (92.1–99.7)	5.0 (−2.1 to 12.5)	0.09 (one-sided)
***P. falciparum,*** ** day 42**	*n*	198	100	98		
	ACPR *n*	184	90	94		
	Percent (95% CI)	92.9 (88.2–95.9)	90.0 (82.0–94.8)	95.9 (89.3–98.7)	5.9 (−2.3 to 14.4)	0.09 (one-sided)
***P. vivax,*** ** day 28**	*N*	47	20	27		
	ACPR *n*	39	13	26		
	Percent (95% CI)	83.0 (68.7–91.9)	65.0 (41.0–83.7)	96.3 (79.1–99.8)	31.3 (5.9 to 55.6)	0.007 (two-sided)
***P. vivax,*** ** day 42**	*N*	47	20	27		
	ACPR *n*	32	6	26		
	Percent (95% CI)	68.1 (52.8–80.5)	30.0 (12.8–54.3)	96.3 (79.1–99.8)	66.3 (36.5 to 83.8)	<0.001 (two-sided)
***P. vivax*** ** after treatment for falciparum malaria, day 42**	*N*	198	100	98		
	ACPR *n*	156	62	94		
	Percent (95% CI)	78.8 (72.3–84.1)	62.0 (51.7–71.4)	95.9 (89.3–98.7)	33.9 (22.5 to 44.6)	<0.001 (one-sided)

**Table 10:** Modified intention-to-treat analysis of treatment response by treatment allocation utilizing a best-case scenario (i.e., all instances of missing data during follow-up scored as parasite-negative).

Endpoint	Number and Category	Total	Artemether-Lumefantrine	Artemisinin-Naphthoquine	Difference (95% CI)	*p*-Value
***P. falciparum,*** ** day 28**	*n*	198	100	98		
	ACPR *n*	197	99	98		
	Percent (95% CI)	99.5 (96.8–100)	99.0 (93.8–100)	100 (95.3–100)	1.0 (−3.8 to 6.2)	0.51 (one-sided)
***P. falciparum,*** ** day 42**	*n*	198	100	98		
	ACPR *n*	196	98	98		
	Percent (95% CI)	99.0 (96.0–99.8)	98.0 (92.3–99.7)	100 (95.3–100)	2.0 (−3.0 to 7.7)	0.25 (one-sided)
***P. vivax,*** ** day 28**	*n*	47	20	27		
	ACPR *n*	40	13	27		
	Percent (95% CI)	84.8 (70.5–93.2)	65.0 (41.0–83.7)	100 (84.5–100)	35.0 (10.7 to 59.1)	0.001 (two-sided)
***P. vivax,*** ** day 42**	*n*	47	20	27		
	ACPR *n*	33	6	27		
	Percent (95% CI)	69.6 (54.1–81.8)	30.0 (12.8–54.3)	100 (84.5–100)	70.0 (41.2 to 87.2)	<0.001 (two-sided)
***P. vivax*** ** after treatment for falciparum malaria, day 42**	*n*	198	100	98		
	ACPR *n*	166	68	98		
	Percent (95% CI)	83.8 (77.8–88.5)	68.0 (57.8–76.8)	100 (95.3–100)	32.0 (22.0 to 42.2)	<0.001 (one-sided)

The two vivax malaria treatment groups were also well matched for baseline characteristics (see Table 5). Parasite clearance was more rapid in children with vivax malaria than in the children with falciparum malaria, with >84% of children with vivax malaria in both treatment groups slide-negative for asexual forms of *P. vivax* by day 1, and 100.0% in both treatment groups both afebrile and aparasitemic by day 3 (see Table 6).

The day-42 PCR-uncorrected ACPR for *P. vivax* infections was 30.0% (95% CI 12.8%–54.2%; six of 20 children) in the artemether-lumefantrine group and 100% (95% CI 84.5%–100%; 26 of 26 children) in the artemisinin-naphthoquine group (see Figure 3 and Table 7). Using these data and the same type 1 error probability and power as in the original sample size estimates, the 46 children who completed the trial exceeded the total of ten children required to show superiority of artemisinin-naphthoquine for this primary endpoint [28]. The significantly greater day-42 ACPR for artemisinin-naphthoquine remained after PCR correction of post-treatment responses for infections with different genetic profiles than those at baseline (100% [95% CI 84.0%–100%] versus 60.0% [95% CI 36.4%–80.0%] in the artemether-lumefantrine-treated children, *p<*0.001; see Table 8). The modified intention-to-treat analyses showed results that were consistent with these findings (see Tables 9 and 10).

The single patient with *P. malariae* infection was randomized to artemether-lumefantrine and responded with prompt parasite clearance and an ACPR at days 28 and 42.

### Gametocyte Carriage and Hemoglobin Concentration

**Figure 4:**
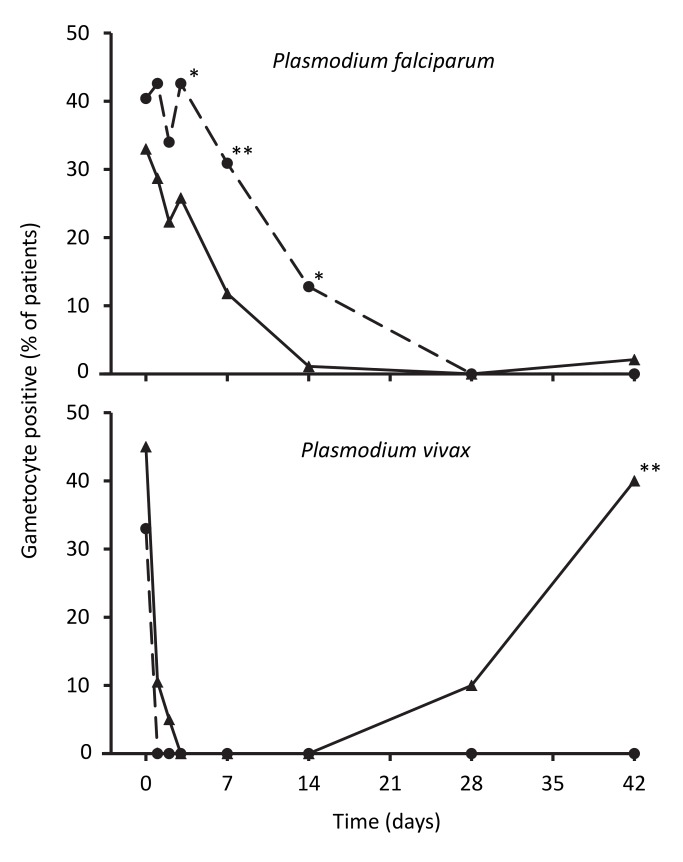
Proportions of patients with gametocytes during follow-up after treatment for *P. falciparum* and for *P. vivax*. Data are for artemether-lumefantrine (▴, solid lines) and artemisinin-naphthoquine (•, dashed lines). *p<0.05, **p<0.01 for between-treatment comparisons.

The percentage of children carrying *P. falciparum* gametocytes based on microscopy was not significantly different between groups at baseline (*p = *0.36), but artemisinin-naphthoquine-treated patients were more likely to have gametocytemia between days 3 and 14 (see Figure 4). The odds ratio for carriage in the artemisinin-naphthoquine group was 3.2 (95% CI 1.5–6.8) that of children in the artemether-lumefantrine group on day 7, and 15.1 (95% CI 1.9–118.1) on day 14. When only those children who were gametocyte positive at baseline were considered (*n = *32 in the artemether-lumefantrine group and *n = *39 in the artemisinin-naphthoquine group), the equivalent odds ratios for gametocyte carriage on days 7 and 14 were 2.6 (95% CI 1.0–6.8) and 10.7 (95% CI 1.3–88.8).

In patients with vivax malaria, there was prompt gametocyte clearance in both groups by day 4, but, consistent with more frequent treatment failure in the artemether-lumefantrine group, a significant proportion of patients (40.0%) had become positive for gametocytes by day 42, while none of the artemisinin-naphthoquine-treated children were gametocytemic at that time (see Figure 4).

**Figure 5:**
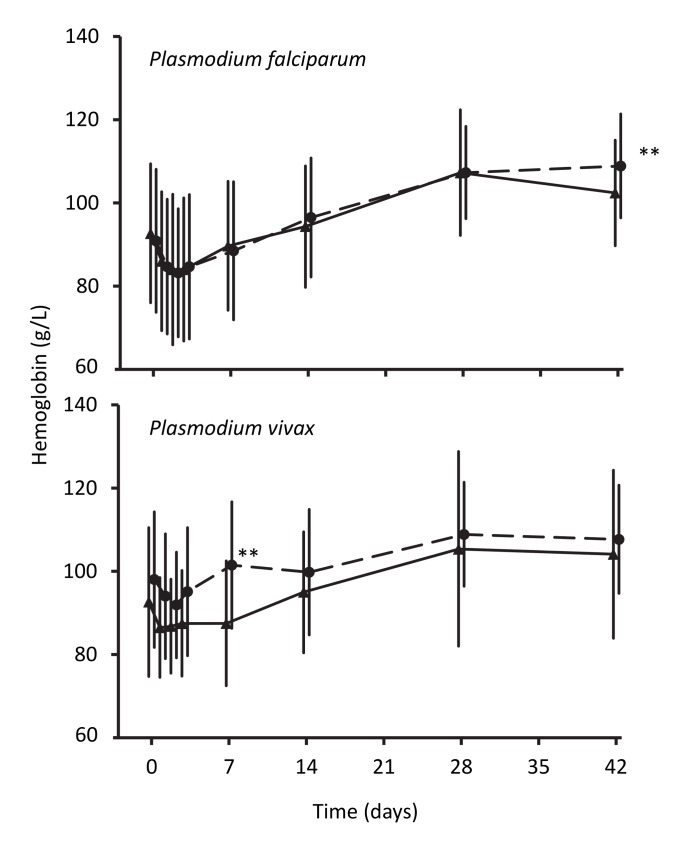
Hemoglobin concentrations during follow-up after treatment for *P. falciparum* and for *P. vivax*. Data are mean and standard deviation (vertical bars) for artemether-lumefantrine (▴, solid lines) and artemisinin-naphthoquine (•, dashed lines). **p<0.01 for between-treatment comparisons.

Hemoglobin concentrations in children treated for falciparum malaria were similar in the two treatment groups until day 42 after treatment, when the artemisinin-naphthoquine-treated children had a significantly higher mean concentration (109±13 g/l versus 102±12 g/l in the artemether-lumefantrine group, *p<*0.001; see Figure 5). In patients with vivax malaria, the hemoglobin concentrations had diverged significantly at day 7, but this difference was not sustained.

## Discussion

Three daily doses of artemisinin-naphthoquine proved a highly efficacious treatment for both falciparum and vivax malaria in PNG children living in an area of intense transmission of multiple *Plasmodium* species. This efficacy profile addresses recognized species-specific shortcomings associated with artemether-lumefantrine and dihydroartemisinin-piperaquine in this patient group [5]. Artemisinin-naphthoquine was well tolerated, and there were no significant safety concerns identified through detailed post-treatment monitoring. Artemisinin-naphthoquine treatment was associated with a higher day-42 hemoglobin concentration than artemether-lumefantrine treatment, with potential implications for attenuated morbidity associated with subsequent malarial episodes and other illnesses. A further advantage of artemisinin-naphthoquine is that it is taken once daily and without the need for co-administration with fat [10], which should improve adherence compared with the relatively complex six-dose artemether-lumefantrine schedule with milk co-administration. Artemisinin-naphthoquine was, however, associated with greater post-treatment gametocyte carriage and, as is the case for a number of other ACT partner drugs, with prolongation of the QT_c_ interval.

There were no reappearances of asexual forms of either *P. falciparum* or *P. vivax* during 42 d of follow-up in any child treated with three daily doses of artemisinin-naphthoquine in the present study. Treatment failures were observed in our previous single- and two-dose studies in PNG children aged 5–10 y [11], and most studies in adults utilizing similar regimens have fallen short of a 100% ACPR, even with only 28 d of follow-up [8],[9],[29],[30], including an Indonesian study with a 28-d ACPR of 79% in mixed *P. falciparum/P. vivax* infections [8],[9]. Only one other study, in Chinese adults with vivax malaria, has utilized a 3-d artemisinin-naphthoquine regimen (17.5 mg/kg artemisinin plus 7 mg/kg naphthoquine versus 20 mg/kg artemisinin plus 8 mg/kg artemisinin in the present study), and this regimen was associated with a day-42 ACPR of 98.4% [31]. Overall, these data support a 3-d regimen to maximize the efficacy of artemisinin-naphthoquine. The present artemisinin-naphthoquine outcomes are clearly superior to the outcomes of both artemether-lumefantrine and dihydroartemisinin-piperaquine in our previous multi-arm trial in young PNG children, since these regimens were associated with *P. falciparum* day-42 PCR-corrected ACPRs of 95.2% and 87.9%, respectively, and *P. vivax* day-42 uncorrected ACPRs of 30.3% and 69.4%, respectively [5].

We hypothesize that the better efficacy of artemisinin-naphthoquine reflects naphthoquine's relatively long terminal elimination half-life and wide therapeutic index. The mean elimination half-life of naphthoquine in PNG children is 21–25 d [10] compared with 14–21 d for piperaquine in this patient group [32]–[34]. This indicates that naphthoquine can suppress reappearances of both *P. falciparum* and *P. vivax* for a longer period than piperaquine and especially lumefantrine, which has an elimination half-life of only 4–5 d [35]. In addition, the magnitude of piperaquine dosing is limited by gastrointestinal side effects including nausea [13]. Currently recommended doses of dihydroartemisinin-piperaquine may be inadequate in young children [36] such as those who participated in our present and previous [5] studies, but higher doses may have reduced tolerability. Gastrointestinal and other symptoms in our artemisinin-naphthoquine-treated children were mild and short-lived despite the use of the manufacturer's recommended single-dose regimen on three consecutive days.

There are theoretical reasons why artemisinin-naphthoquine may be a suboptimal ACT. As shown by ourselves [10] and others [37], auto-induction of artemisinin metabolism results in declining plasma concentrations over the 3 d of treatment. Artemether also induces its own metabolism [38], but unlike artemisinin, which has an inactive metabolite, artemether is converted to dihydroartemisinin, which has the greatest activity of all derivatives against *P. falciparum* [39]. These pharmacokinetic/pharmacodynamic factors reflect the relatively low efficacy of artemisinin-naphthoquine given for less than 3 d [8],[9],[11] and may have contributed to the greater percentage of gametocytemic children during the first 2 wk after artemisinin-naphthoquine treatment in the present study. The persistence of *P. falciparum* gametocytemia has implications for malaria control and could be managed by administration of single-dose primaquine, provided that cost-effective point-of-care screening for glucose-6-phosphate dehydrogenase deficiency is available [40]. It is possible that the need for longer courses of primaquine for radical cure of *P. vivax* may be less with artemisinin-naphthoquine given the lack of appearance of asexual forms of this species during the 42-d follow-up period in children treated for acute malaria with this ACT, but this possibility needs to be evaluated in further studies with longer-term follow-up.

Despite potential pharmacokinetic/pharmacodynamic shortcomings, initial asexual parasite clearance was as rapid with artemisinin-naphthoquine as with artemether-lumefantrine. There is in vitro evidence of cross-resistance between chloroquine and naphthoquine in *P. falciparum* isolates from PNG, as is also the case for piperaquine [41]. Whether this has clinical implications for recrudescence in areas of chloroquine-resistant *P. falciparum* and *P. vivax* is uncertain, but it did not appear influential in the present study, even though the majority of *P. falciparum* strains in Madang Province (>80%) show in vitro evidence of chloroquine resistance with near fixation of the resistance-associated *pfcrt* allele [40]. The three-dose artemisinin-naphthoquine combination regimen that complied with WHO recommendations for all ACTs [12] and that was developed for the present study to maximize efficacy against asexual and sexual parasite forms [11] could have unmasked significant adverse effects such as the hepatotoxicity that prevented inclusion of artesunate-pyronaridine in the present study [16],[17],[42], but the present safety data are reassuring.

Naphthoquine is a tetra-aminoquinoline drug, and our previous preliminary safety study of artemisinin-naphthoquine in older PNG children did not find evidence of aminoquinoline-specific side effects such as hearing loss, orthostatic hypotension, and hypoglycemia [11]. Similarly, the artemisinin-naphthoquine-treated children in the present study did not complain of deafness, tinnitus, dizziness, or posture-related symptoms during follow-up, and no cases of hypoglycemia were recorded. The lack of hematologic and hepatorenal toxicity in the present study is also consistent with our previous findings [11]. An important outcome variable in the present study was electrocardiographic effects, and there was significant QT_c_ prolongation between baseline and 4 h after the final dose in our artemisinin-naphthoquine-treated children. Since there were no significant between-group differences in pulse rate and fever clearance during initial monitoring, this QT_c_ prolongation is consistent with a naphthoquine-specific effect rather than reflecting a disproportionately larger reduction in sympathetic activity with recovery in artemisinin-naphthoquine-treated children that could have independently promoted an increase in the QT_c_ interval in this group [43].

Many aminoquinoline drugs cause QT_c_ prolongation. Chloroquine increases the QT_c_ by a mean of up to 30 msec^0.5^ in healthy volunteers [44], but no malignant arrhythmias or sudden deaths have been reported with recommended antimalarial doses [43]. Piperaquine also has this effect, with studies in children in PNG and Cambodia showing QT_c_ prolongation similar to that in the present study for children treated with artemisinin-naphthoquine [19],[20]. Repeated piperaquine dosing with the potential for accumulation is not associated with definite cardiotoxicity [45], and there is no in vitro evidence of torsadogenic or other pro-arrhythmic effects [46]. In the case of halofantrine (now withdrawn), cardiotoxicity is plasma-concentration-dependent [43], but the day-2 QT_c_ prolongation observed in our artemisinin-naphthoquine-treated children had resolved by day 7, when plasma naphthoquine concentrations would still have been high [10].

This latter observation, amongst other considerations, suggests that the artemisinin-naphthoquine regimen used in the present study was not pro-arrhythmic. Relatively frequent QT_c_ prolongation has been observed in young children presenting with untreated malaria [43] and other serious illnesses [47], but it is transient and not associated with adverse outcomes. There has been a lack of clinically evident cardiotoxicity in the present and other studies of naphthoquine [8], including three daily doses for vivax malaria in adults [31]. Nevertheless, given concerns that piperaquine-containing ACTs should not be used in people who have, or are at risk of, QT_c_ prolongation or cardiac arrhythmias (including those with electrolyte disturbance such as hypokalemia and hypocalcemia), and that they should not be taken with other drugs that prolong the QT_c_ interval [48], it may be appropriate to recommend similar restrictions for three-dose artemisinin-naphthoquine regimens.

Our study had limitations. Baseline microscopy by study staff under field conditions led to 11% of the 267 randomized patients being included when they had sub-threshold parasitemia based on expert microscopy, while electrocardiography proved difficult in a significant minority of children. We did not directly observe all artemether-lumefantrine doses, but parents/guardians were instructed as to the importance of all doses given at home, adherence was assessed at the next follow-up visit, and day-7 plasma lumefantrine concentrations were consistent with full adherence in each case. The children screened and recruited were drawn from coastal PNG communities that are typical of those in Oceania where hyper- or holo-endemic transmission of multiple *Plasmodium* species is found. It is possible that treatment responses in children with uncomplicated malaria who have been raised in areas with lower-level transmission may be attenuated because of their reduced or absent malarial immunity. Nevertheless, ACTs such as artemether-lumefantrine are considered efficacious where there is unstable transmission [12], while most of the studies evaluating artemisinin-naphthoquine—which had mostly acceptable efficacy—were conducted in this epidemiologic setting [8],[9]. The strengths of the study are the high rates of laboratory (including parasitologic) data collection and the ≥92% retention rate of children in each of the four species/treatment arms, which was greater than anticipated when sample sizes were determined and greater than in our previous multi-arm trial in PNG children (with, for example, only 77% in one of the arms [5]).

The efficacy, tolerability, and safety of three daily doses of artemisinin-naphthoquine suggest that this regimen should be considered together with other currently available effective ACTs for treatment of uncomplicated malaria in PNG and similar epidemiologic settings with transmission of multiple *Plasmodium* species. Artemisinin-naphthoquine may have advantages where *P. vivax* predominates. Although pharmaco-economic analyses were beyond the scope of the present study, it seems likely that the additional cost of three doses compared to the single dose currently recommended will be more than offset by the substantial reduction in post-treatment vivax malaria.

## Supporting Information

Text S1 **Consort checklist for a randomized clinical trial of artemisinin-naphthoquine versus artemether-lumefantrine for uncomplicated malaria in Papua New Guinean children.**
(DOC)

Text S2 **Protocol for the clinical trial “Novel artemisinin-based combination therapies for Papuan New Guinean children exposed to high transmission of multiple Plasmodium species.”**
(DOC)

## Abbreviations

ACT: artemisinin combination therapy
ACPR: adequate clinical and parasitologic response
ALT: alanine aminotransferase
ETF: early treatment failure
FCT: fever clearance time
LCF: late clinical failure
LPF: late parasitologic failure
PNG: Papua New Guinea
PCR: polymerase chain reaction
PCT: parasite clearance time
QT_c_: electrocardiographic QT interval corrected for heart rate
ULRR: upper limit of the reference range
WHO: World Health Organization
