# Supplementary material for: Artemisinin-Naphthoquine versus Artemether-Lumefantrine for Uncomplicated Malaria in Papua New Guinean Children: An Open-Label Randomized Trial
Source: PLoS Med. 2014 Dec 30;11(12):e1001773. doi: 10.1371/journal.pmed.1001773 (PMC4280121; doi:10.1371/journal.pmed.1001773)
Supplement: Text S2 — Protocol for the clinical trial “Novel artemisinin-based combination therapies for Papuan New Guinean children exposed to high transmission of multiple Plasmodium species.” (DOC) [file pmed.1001773.s002.doc]

**Text S2**

**PARTICIPATING INSTITUTIONS:**

1. Papua New Guinea Institute of Medical Research (PNGIMR)

2. University of Western Australia (UWA)

3. Curtin University (CU)

4. Divine Word University (DWU)

**PRINCIPAL INVESTIGATORS:**

Professor Tim Davis (UWA)

Dr Ivo Mueller (PNGIMR)

Professor Peter Siba (PNGIMR)

Associate Professor Kevin Batty (CU)

Dr Harin Karunajeewa (UWA)

Professor Tim St Pierre (UWA)

**ASSOCIATE INVESTIGATORS:**

Professor Francis Hombanje (DWU)

Dr Moses Laman (PNGIMR)

Ms Brioni Moore (UWA)

Dr Inoni Betuela (PNGIMR)

Professor Ken Ilett (UWA)

Dr John Benjamin (PNGIMR)

**FUNDING BODY:**

National Health and Medical Research Council of Australia

**AIMS AND HYPOTHESES**

**Primary aim:**

To conduct a 3-year open-label, randomized, parallel-group trial of the tolerability, safety, efficacy and cost-effectiveness of the new artemisinin combination therapies (ACTs) artesunate-pyronaridine (AP) and artemisinin-naphthoquine (AN) compared with standard artemether-lumefantrine (AL) therapy in Papua New Guinean (PNG) children with *Plasmodium falciparum* or *P. vivax* infections.

**Secondary aims:**

1. To determine, using sensitive magnetic methods, the effect of each ACT regimen on gametocyte carriage during the six-week trial follow-up period.

2. To determine the duration of post-treatment prophylaxis using passive case detection of symptomatic malaria infections presenting over a period of six months after trial follow-up.

**BACKGROUND**

In PNG, malaria is highly endemic in most coastal and island regions where the majority of people live. There are >1.5 million suspected cases annually (equivalent to >25% of the population), and malaria is the second-leading cause of both hospitalisations and death.^1,2^ Most morbidity and fatalities occur in young children with *P. falciparum* infections. However, although vivax malaria has been considered ‘benign’, recent studies from PNG^3^ and elsewhere^4^ have highlighted the potential for complications and even death. Of the four species of *Plasmodium* found in PNG, *P. falciparum* infections are the most prevalent but *P. vivax* transmission is probably the highest in the world.^5^

The cornerstone of strategies for reducing malaria-related morbidity and mortality is access to prompt, effective and affordable treatment, particularly for children who are most at risk of complications and death. The malaria situation in countries in Oceania including PNG parallels that in sub-Saharan Africa where there are likely to be dire consequences if the use of increasingly ineffective conventional antimalarial drugs such as chloroquine (CQ) and sulfadoxine-pyrimethamine (SP) continues.^6^ The WHO now recommends that ACT is used first-line for uncomplicated malaria, with the choice between available ACTs guided by local parasite resistance to the longer half-life partner drug.^7^

We recently conducted a comparative trial of conventional CQ-SP and the ACTs artesunate-SP, AL and dihydroartemisinin-piperaquine (DP) in children aged 0.5-5 years from Madang or Kunjingini with microscopically confirmed falciparum or vivax malaria, the results of which were published recently in the *New England Journal of Medicine*.^8^ The most effective treatment for *P. falciparum* was AL which had a 95.2% PCR-confirmed adequate clinical and parasitological response (ACPR) at 42 days compared with 81.5% in CQ-SP, 85.4% in artesunate-SP and 88.0% in dihydroartemisinin (DHA)-piperaquine (DP) treated patients. AL was, therefore, the only treatment to fulfil WHO requirements for prospective new therapies for falciparum malaria (>95% ACPR during follow-up of ≥28 days’ duration^7^). However, the highest 42-day *P. vivax* ACPR was in DP treated patients (69.4% vs ≤33.3% in the other three arms).

These data suggest that AL and DP were the most efficacious regimens against *P. falciparum* and *P. vivax*, respectively, in PNG children. However, neither is optimal where there is intense transmission of multiple *Plasmodium* species, an epidemiological context that is likely to apply in other countries in Oceania and South-east Asia. One approach to finding more broadly efficacious treatments in this situation is to assess alternative ACTs with new partner drugs. Two such ACTs at a relatively advanced stage of clinical development are artesunate-pyronaridine (AP; Pyramax, Shin Poong Pharm. Inc., Seoul, Korea) and artemisinin-naphthoquine (AN; Arco, Kunming Pharmaceutical Corp., Yunnan, China).

AP is a fixed-dose tablet/granule co-formulation. Artesunate has, based on our own studies,^9, 10^ the most favourable pharmacological properties of the orally-administered artemisinin derivatives. Pyronaridine phosphate (a naphthyridine derivative used in China since the 1980’s)^11^ has proved effective as monotherapy in areas with CQ-resistant *P. falciparum*.^12-14^ While the disposition of artesunate is well characterised, there are limited data on pyronaridine pharmacokinetics. Dose-ranging studies employing three daily doses^15^ and early data from China^11^ suggest that it is promptly absorbed and has a terminal elimination half-life (t_½_*_e_*) of 7-9 days. Most of its adverse effects are gastrointestinal and mild^11, 15^ and there are no changes in haematological or biochemical test results after treatment.^15^ A suite of additional pharmacological, efficacy and safety studies in selected (non-Melanesian) groups of adults and children with falciparum malaria has been completed and the data should be published shortly (see <http://clinicaltrials.gov/ct2/results?term=pyronaridine>).

AN is a fixed-dose tablet co-formulation that is marketed commercially in PNG and other countries. It is recommended as single-dose treatment even though WHO stipulates that ACTs should be give as at least a three-day regimen.^7^ As with AP, the pharmacokinetics of the artemisinin component are well characterised but there are very few published data for naphthoquine. Naphthoquine appears to be promptly absorbed with a t_½_*_e_* that has been reported as between 2 days^16^ and 12 days (Arco Product Information). Available data from China^16^ and Indochina^17^ suggest that AN is effective and well-tolerated.

Unfortunately, our own as yet unpublished pharmacokinetic and efficacy data from 47 PNG children raise concerns regarding the efficacy of single-dose AN. There is a high rate of malaria slide positivity on the third day of treatment (>25%) and gametocyte carriage is also much higher than with AL and DP.^8^ This relates to the use of only 20 mg/kg artemisinin in the single dose. Conventional dosing requires a further 2-4 days of at least 10 mg artemisinin/kg.^18^ We have been conducting preliminary pharmacokinetic studies in these children to determine whether a three-day AN regimen could be safe and effective. Plasma concentrations derived using a validated hplc assay are shown in the Figure below for two full recommended doses given 24 hours apart. The data suggest that, due to a brisk post-dose distribution phase and the age of the patients (children requiring sometimes substantially greater mg/kg doses of antimalarial drugs than adults to achieve adequate plasma concentrations), the two-dose regimen gives peak plasma naphthoquine concentrations (C_max_) that are still below those reported in adults after a single 9 mg/kg dose (99-245 μg/L)^16^ and was not associated with any toxicity including symptoms such as nausea, headache and dizziness, ECG changes, postural hypotension or hearing loss. Furthermore, pharmacokinetic modelling predicts that 3 daily doses would be very unlikely to exceed the reported C_max_ upper limit of 245 μg/L that has been reported in adults. This three-dose regimen provides a total of 60 mg/kg artemisinin which approaches that recommended for treatment of falciparum malaria.^18^ In addition, there will be effective concentrations of naphthoquine present for at least 3 weeks after treatment (see Figure).

**Figure.** Plasma concentration-time profile for naphthoquine from 10 PNG children given two doses of artemisinin-naphthoquine (Arco®; 2 × 23 and 9 mg/kg) for treatment of uncomplicated falciparum malaria. Solid line represents the two-compartment model fitted to the plasma naphthoquine concentrations (mean ± SD).

This application outlines studies designed to provide robust data on the efficacy of the novel ACTs AP and AN in comparison with first-line AL in PNG children. There will be particular interest in the relative efficacy of these regimens against *P. vivax* given the low ACPR we observed previously.^8^

**RESEARCH PLAN**

We will perform the clinical studies at i) Alexishafen and Mugil Clinics, Madang Province, sites we have used for various studies since 2001 and which have proven recruitment potential and excellent facilities,^CIA#31^ and ii) Vunapope Hospital, East New Britain Province which also has a busy clinical load and laboratories which have been used by the PNG Institute of Medical Research (IMR) for malaria studies. Maprik in the East Sepik may be brought in as an additional site depending on recruitment. Laboratory studies (host/parasite genotyping, drug assays and/or biochemical testing) will be conducted at Vunapope; the IMR laboratories at Yagaum Hospital, Madang Province; the University of Western Australia (UWA) School of Medicine and Pharmacology and the Biochemistry Department at Fremantle Hospital; the Pharmacy School, Curtin University. Relevant techniques are well established in these institutions.

**Main Study: A trial of the tolerability, safety, efficacy and cost-effectiveness of AL, AP and AN in PNG children with uncomplicated falciparum or vivax malaria** *(Primary aim)*

*Background:* Since pyronaridine^11-14^ and naphthoquine^16^ are active against CQ-resistant falciparum malaria and have a t_½_*_e_* that is at least that of lumefantrine (4-6 days), AP and AN should have similar efficacy over 42 days of follow-up to AL in this situation. We postulated that the greater efficacy of DP vs AL against *P. vivax* reflects, in part, the longer piperaquine t_½_*_e_* (3-4 weeks), in which case the efficacy of AP and perhaps AN should equate with that of DP. However, the similar low efficacy of AL and CQ-SP in areas with known CQ-resistant *P. vivax* means that other factors may underlie the poor response to AL in our recent trial.^8^

*Operational principles:* We will conduct the study using standard operating procedures, under Good Clinical Practice guidelines, in accordance with the Helsinki Declaration, and with ethical approval from the PNG Ministry of Health Medical Research Advisory Committee and the UWA Human Research Ethics Committee. Although the trial is open-label, there will be an independent Data and Safety Monitoring Committee (DSMC) and external monitoring of sites.

*Endpoints:* The primary endpoint will be:

1. recrudescent *P. falciparum* within 42 days after correction for re-infections using PCR genotyping of polymorphic parasite loci^19, 20^ in children with falciparum malaria, or
2. appearance of any *P. vivax* parasitaemia within 42 days after treatment for vivax malaria.

Secondary endpoints in falciparum malaria will be:

1. reappearance of PCR-uncorrected *P. falciparum* parasitemia,
2. appearance of any *P. vivax* parasitemia, and
3. appearance of *P. falciparum* gametocytes, all within 42 days.

Secondary endpoints in vivax malaria will be:

1. reappearance of any *P. vivax* parasitemia within 28 days, and
2. appearance of *P. vivax* gametocytes within 42 days.

Other secondary endpoints for each *Plasmodium* species will be initial fever and parasite clearance times (the times to the first of two consecutive assessments at which the child was afebrile and slide-negative, respectively), and drug safety including haemoglobin levels.

*Sample sizes:* Sample size calculations^21^ for the present trial are based on the assumptions that i) the *P. falciparum* Day 42 PCR-corrected ACPR for AL is 95.2%^8^ with a non-inferiority margin of 5% for AP and AN, ii) the *P. vivax* Day 42 uncorrected ACPR for AP and AN is at least double the 30.3% found previously for AL (as for DP in the original trial^8^), iii) there will be equal numbers in each treatment arm, and iv) there will be a 25% attrition rate:

| Study | Type | Total *n* | *n*/group with drop-outs | Power | Α |
| --- | --- | --- | --- | --- | --- |
| *P. falciparum* | Non-inferiority | 515 | 220 | >80% | 0.05 (one-tailed) |
| *P. vivax* | Superiority | 122 | 60 | >90% | 0.05 (two-tailed) |

*Subjects:* Children aged 0.5-5 years with an axillary temperature >37.5°C or fever during the previous 24 hours will be screened using on-site blood film microscopy. Those with either *P. falciparum* (>1000 asexual parasites/μL whole blood) or *P. vivax* (>250/μL) will be eligible if they have i) no features of severity,^22^ ii) not taken a study drug in the previous 28 days, iii) no clinical (including anthropometric) or laboratory evidence of another infection or co-morbidity including malnutrition. Informed consent will be obtained from parents/guardians.

*Clinical/laboratory procedures:* An initial (Day 0) history will be taken and physical examination performed. Blood will be taken for measurement of haemoglobin and glucose. Treatment allocation will be by computer-generated randomized design in blocks of 24 by site:

1. AL (Novartis Pharma, Basel, Switzerland) as artemether 1.7 mg/kg and lumefantrine 10 mg/kg twice-daily for three days,
2. AP (Pyramax) as artesunate 3 mg/kg and pyronaridine tetraphosphate 9 mg/kg once-daily for three days, and
3. AN (Arco) as artemisinin 20 mg/kg and naphthoquine phosphate 8 mg/kg once-daily for three days (as justified above).

Combinations of full, half- or quarter-tablets will be swallowed whole or crushed lightly before administration. AL-treated subjects will be given tablets or suspension with milk; because there is no such recommendation for AP or AN, pharmacokinetic studies our group will perform during 2009 will determine whether these ACTs should also be given with milk. Only the evening AL doses will be unsupervised, being given at home by parents/guardians. Children vomiting within 30 minutes will be re-treated.

Standardized review including microscopy will be scheduled on Days 1, 2, 3, 7, 14, 28 and 42, with all data, including symptoms, unscheduled clinic visits and non-study treatment, entered on detailed case report forms. Children developing uncomplicated or severe malaria will be given DP at the clinic or intramuscular artemether at the local referral hospital, respectively as per the most recent PNG National Treatment Guidelines.^23^ All blood films will be re-examined by two skilled microscopists blinded to treatment. Slides discrepant for positivity/negativity, speciation or parasitaemia (>3x difference) will be adjudicated by a senior microscopist. The primary endpoints constitute ACPR under WHO-recommended assessment.^24^ Early treatment failure (ETF) will be taken as the development of severity or an inadequate parasitological response by Day 3. Any child developing parasitaemia between Days 4 and 42 will be considered late parasitological failure (LPF) or, if febrile, late clinical failure (LCF).

At baseline and each subsequent visit, a finger-prick blood smear and filter paper blood spot will be taken, in addition to a 250 μL mixed capillary EDTA sample which will be centrifuged and the plasma separated. In addition, a 3-5 mL blood sample will be taken by venepuncture at baseline, day 7 and 28 for antimalarial drug assay. If a child presents with fever or has a positive blood film for malaria at other times, a single blood sample will also be taken – this will normally replace the day 28 sample if there is a re-infection/recrudescence which means the child has reached a pre-specified endpoint. The total volume of blood taken from any child will be <15 mL over 6 weeks. Although there is evidence that Day 7 plasma drug concentrations are independently associated with response for lumefantrine- and PQ-based ACT,^25^ there has been no study that has looked at pyronaridine or naphthoquine, or simultaneously incorporated other potentially important variables such as baseline *P. falciparum* parasitaemia in predictive models. Our finding that baseline parasitaemia predicted outcome in DP-treated patients^8^ should also be assessed simultaneously with other parasite- and drug-specific factors in a large patient sample and using other long-half life partner drugs. We did not identify anthropometric features that predicted outcome in our previous trial and postulate the same result with the newer regimens. Plasma drug concentrations at the time of treatment failure have been used qualitatively to identify resistant parasites, but no formal *in vivo* MIC estimate the has been derived.^25^

We have developed high performance liquid chromatography assays for lumefantrine, naphthoquine and pyronaridine, but now have access to liquid chromatography-mass spectrometry which allows greater assay sensitivity with a smaller sample volume and increased throughput. Day 7 and follow-up samples on the day of reappearance of parasitaemia will be taken from trial patients, centrifuged promptly, and separated plasma kept and transported frozen until assayed at Curtin University. Batches of each treatment will also be retained for assay of content. We have *in vitro* parasite culture facilities at Yagaum and these can be set up at Vunapope. We will utilise the *Plasmodium* lactate dehydrogenase colorimetric assay for drug sensitivity testing.

*Interim analysis:* Although the AN regimen to be used in the trial has been developed using detailed efficacy and safety assessment, a validated assay and conventional pharmacokinetic modelling in PNG children, we will perform an interim analysis after the first 50 children have received this form of treatment. This analysis will be done by the Data and Safety Monitoring Committee (Prof John Vince (UPNG), Dr Laurens Manning (UWA) and Dr Wendy Davis UWA – statistician) who will decide whether the AN arm should be retained for the rest of the trial. The criteria to be used will be i) day 3 parasitaemia positive >25% of children, ii) gametocyte carriage >25% on day 7 and/or iii) >25% attrition rate by day 7.

If AN is withdrawn, children in this arm will be switched to DP (dihydroartemisinin 2.5 mg/kg and piperaquine phosphate 20 mg/kg daily for three days; Sigma Tau, Rome, Italy) given with milk at each dose. DP was one of the arms in our original treatment trial^8^ and is currently recommended second-line treatment in PNG for uncomplicated malaria in children. However, there has been the suggestion that cure rates might improve if fat were co-administered with this form of ACT to enhance bioavailability,^28^ as it is with AL. As per the Manufacturer’s instructions, DP was administered with water in the original trial.^8^ All study procedures will remain the same for children in this group.

*Data analysis:* Statistical analysis will be by *a priori* plan. Per-protocol analyses will include children with complete follow-up or a confirmed treatment failure, and will exclude those treated for malaria without confirmatory microscopy or who defaulted from follow-up despite repeated attempts at contact. These excluded patients will be retained in modified intention-to-treat analyses utilizing:

1. a worst-case approach (ETF assumed for Day 3 exclusions, LPF/LCF otherwise) and
2. a best-case approach (all missing blood films assumed parasite-negative).^8^

Kaplan-Meier estimates will be computed for each endpoint by parasite species. Treatments will be compared by log-rank test. Safety and tolerability will be assessed from the incidence of symptoms/signs to Day 7 using Poisson regression (frequent events) or Fisher’s exact test (infrequent events). Blinded interim efficacy analyses and safety assessments will be performed under the auspices of the DSMC and the trial terminated prematurely if one treatment violates the non-inferiority margin or unexpected toxicity emerges. Comprehensive health care cost data will be collected for each patient from Day 0 to Day 42 as is being done currently for the previous trial,^8^ but indirect costs (e.g. reduced parental/guardian income due to the child’s illness) will also be ascertained in the present trial.

Cox regression using backward-stepwise modelling will be used to determine predictors of treatment failure among age, sex, measures of growth/nutrition, baseline haemoglobin, baseline parasitaemia, Day 7 partner drug concentrations and drug-specific IC_50_ values. We will utilise population pharmacokinetic modelling using NONMEM (Project Group, University of California San Francisco) and models of *P. falciparum* parasite development based on our animal studies to estimate MIC and derive secondary variables of clinical importance such as the time, in 48-hour parasite life-cycles, above MIC. We have performed host genotyping for red cell traits including thalassemia, South-east Asian ovalocytosis and Gerbich antigen blood group as predictors of response in previous studies and may, if other relevant pharmacogenetic data emerge, extend the proposed analyses to include other polymorphisms.

*Duration/feasibility:* The trial will run from June 2010 to June 2013. We recruited 742 eligible children at Alexishafen and Kunjingini in the East Sepik over 2.3 years in the initial trial,^8^ and foresee no difficulties in recruiting 870 children to the present study over 3 years. The longer time period for the new study allows for the effect of reduced local transmission when AL is introduced.

**Sub-study 1: Effect of treatment on gametocyte carriage during follow-up** *(Secondary Aim 3)*

*Hypotheses:* i) High field gradient magnetic fractionation (HFGMF) can be used to develop a simple but sensitive field test for gametocyte transmission; ii) AL, AP and AN have similar effects on gametocyte carriage for both *P. falciparum* and *P. vivax* infections.

*Background:* We have developed HFGMF gametocyte detection based on the unique metabolism of iron in the parasite and commercially available magnetic separation columns (Miltenyi Biotech). The method relies on magnetic concentration of gametocytes which are then identified more readily on a smear (see Figure 2) than by conventional expert microscopy.^29^ It has a similar sensitivity to reverse transcriptase PCR (RTPCR, data submitted; see Table), but is much quicker and cheaper.


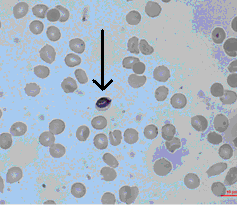


| Gametocyte density | HFGMF (time taken) | Thick film (time taken) | RT-PCR |
| --- | --- | --- | --- |
| 2,970/µL | Positive (<1 minute) | Positive (<1 minute) | Positive |
| 297/µL | Positive (<1 minute) | Positive (7 minutes) | Positive |
| 29.7/µL | Positive (<1 minute) | Positive (28 minutes) | Positive |
| 2.97/µL | Positive (9 minutes) | Negative | Positive |
| 0.297/µL | Negative | Negative | Negative  HFGMF detection at 3/μL |

The method could be used to quantify the gametocidal efficacy of ACT assuming that transmission is unlikely at a gametocyte density <3/μL,^30^ a threshold detectable by HFGMF but not microscopy.

*Methods:* We will take a small additional sample of mixed capillary blood at each follow-up visit from 25 children in each treatment and species arm on which HFGMF and RTPCR will be performed as well as conventional microscopy. The differences in gametocyte carriage by detection method over time between treatment arms will be determined using General Linear Modelling for repeated measures, applying square-root transformation to allow for the effect of zero values on the density distribution at each time point and identifying important non-treatment-related covariates.

**Sub-study 2: Duration of post-treatment prophylaxis using passive case detection of symptomatic malaria during the six months after trial follow-up** *(Secondary Aim 5)*

*Hypothesis:* The duration of post-treatment prophylaxis for AL, AP and AN is a function of the t_½_*_e_* of the non-artemisinin partner drug alone.

*Background:* For some long half-life drugs such as piperaquine, urinary excretion studies suggest unexpected persistence of low plasma concentrations.^31^ In addition, the antimalarial activity of atovaquone in sera is sustained beyond that predicted by its pharmacokinetic properties.^32^ Whether these sorts of effects are of clinical importance in ACT-treated patients is unknown, even for DP.

*Methods:* We will carry out a 6-month period of passive case detection of all trial patients with an ACPR after trial close-out through surveillance of clinic/inpatient records for confirmed malarial episodes at i) Alexishafen/Modilon Hospital and ii) Rabaul Hospital/neighbouring clinics. Our established presence at both sites and our relationship with local health care staff should enable us to overcome potential problems with ascertainment using existing information systems. To examine the effect of treatment on long-term outcome, we will perform a Cox regression analysis of time to first appearance of i) any parasitaemia in-trial or post-trial, and ii) because LPF would not be ascertained during post-trial follow-up, any symptomatic parasitaemia in-trial or post-trial. The mean time to event in each treatment group will be compared with the t_½_*_e_* for each scenario.

**ETHICAL ISSUES**

*i) Administration of a non-standard treatment for malaria:*  Two-thirds of the children will receive a non-standard treatment for malaria. However, both AP and AN have been well tested and shown to be fully effective in a variety of other countries and AN is already available in PNG. It is important for future antimalarial treatment policy to have up-to-date data from PNG children on alternative therapies if combinations such as AL start to fail.

*ii) Informed consent and child assent:* The parents/guardians of all participants will give informed verbal and written consent. Children who are able to understand and communicate at an appropriate level will be given a basic outline of the study and will not be recruited if there is clear evidence of non-assent to study procedures.

*iii) Painful and unpleasant procedures (finger pricks, venesection):* These may cause a small amount of pain and bruising. The risk of infection is negligible.

*iv) Volume of blood sampling:* The total volume of blood taken over 6 weeks will be <15mL, representing approximately 2% of a child’s haematocrit. This is not a physiologically significant amount and it should be replaced quickly after successful treatment of malaria.

*v) Arrangements for adverse events during the trial:* Trained health care staff will be at each site during working hours and contactable by mobile phone out of hours if necessary, and a physician will be either on site or also available for telephone consultation if a child develops or presents with any adverse event. If the adverse event is considered severe,

1. the child will be transferred promptly to the nearest major hospital for further management
2. the principal investigators will be informed of the nature of the event within 24 hours
3. the local study physicians and principal investigators will determine whether it is not related, possibly related or definitely related to allocated treatment, or unknown
4. the principal investigators will notify each event to the Data and Safety Monitoring Committee who will consider whether the protocol should be modified as a result of such events. The PNG IMR Institutional Review Board will also be notified promptly of such events using the standard format for such occurrences.

**REFERENCES**

1. Department of Health. National Health Plan: 2001-2010. Port Moresby: Government of Papua New Guinea; 2000.

2. Naraqi S, Feling B, Leeder SR. Disease and death in Papua New Guinea. Med J Aust 2003;178:7-8.

3. Genton B, D'Acremont V, Rare L, et al. *Plasmodium vivax* and mixed infections are associated with severe malaria in children: a prospective cohort study from Papua New Guinea. PLoS Med 2008;5:e127.

4. Tjitra E, Anstey NM, Sugiarto P, et al. Multidrug-resistant *Plasmodium vivax* associated with severe and fatal malaria: a prospective study in Papua, Indonesia. PLoS Med 2008;5:e128.

5. Muller I, Bockarie M, Alpers M, Smith T. The epidemiology of malaria in Papua New Guinea. Trends Parasitol 2003;19:253-9.

6. White NJ, Nosten F, Looareesuwan S, et al. Averting a malaria disaster. Lancet 1999;353:1965-7.

7. World Health Organization. Guidelines for the treatment of malaria. Geneva: World Health Organization; 2006.

8. Karunajeewa HA, Mueller I, Senn M, Lin E, Law I, Gomorrai PS, Oa O, Griffin S, Kotab K, Suano P, Tarongka N, Ura A, Lautu D, Page-Sharp M, Wong R, Salman S, Siba P, Ilett KF, Davis TME. A trial of combination antimalarial therapies in Papua New Guinean children. New England Journal of Medicine 2008;359:2544-255714.

9. Batty KT, Ilett KE, Powell SM, Martin J, Davis TM. Relative bioavailability of artesunate and dihydroartemisinin: investigations in the isolated perfused rat liver and in healthy Caucasian volunteers. Am J Trop Med Hyg 2002;66:130-6.

10. Binh TQ, Ilett KF, Batty KT, et al. Oral bioavailability of dihydroartemisinin in Vietnamese volunteers and in patients with falciparum malaria. Br J Clin Pharmacol 2001;51:541-6.

11. Shao BR. A review of antimalarial drug pyronaridine. Chin Med J (Engl) 1990;103:428-34.

12. Looareesuwan S, Kyle DE, Viravan C, et al. Clinical study of pyronaridine for the treatment of acute uncomplicated falciparum malaria in Thailand. Am J Trop Med Hyg 1996;54:205-9.

13. Ringwald P, Bickii J, Basco L. Randomised trial of pyronaridine versus chloroquine for acute uncomplicated falciparum malaria in Africa. Lancet 1996;347:24-8.

14. Ringwald P, Bickii J, Basco LK. Efficacy of oral pyronaridine for the treatment of acute uncomplicated falciparum malaria in African children. Clin Infect Dis 1998;26:946-53.

15. Ramharter M, Kurth F, Schreier AC, et al. Fixed-dose pyronaridine-artesunate combination for treatment of uncomplicated falciparum malaria in pediatric patients in Gabon. J Infect Dis 2008;198:911-9.

16. Wang JY, Cao WC, Shan CQ, Zhang M, Li GF, Ding DB, et al. Naphthoquine phosphate and its combination with artemisinine. Acta Trop 2004;89:375-81.

17. Krudsood S, Chalermrut K, Pengruksa C, et al. Comparative clinical trial of two-fixed combinations dihydroartemisinin-napthoquine-trimethoprim and artemether-lumefantrine in the treatment of acute uncomplicated falciparum malaria in Thailand. SE Asian J Trop Med Publ Hlth 2003;34:316-21.

18. German PI, Aweeka FT. Clinical pharmacology of artemisinin-based combination therapies. Clin Pharmacokinet 2008;47:91-102

19. Cattamanchi A, Kyabayinze D, Hubbard A, Rosenthal PJ, Dorsey G. Distinguishing recrudescence from reinfection in a longitudinal antimalarial drug efficacy study: comparison of results based on genotyping of MSP-1, MSP-2, and GLURP. Am J Trop Med Hyg 2003;68:133-9.

20. Felger I, Beck H. Genotyping of *Plasmodium falciparum.* Methods Mol Med 2002;72:117-29.

21. EAST v5. Software for Advanced Clinical Trial Design, Simulation and Monitoring. Cytel Statistical & Services CI. Cambridge MA; 2008.

22. World Health Organisation. Severe falciparum malaria. Transactions of the Royal Society of Tropical Medicine and Hygiene 2000;94 S 1:1-90.

23. Papua New Guinea Department of Health. Standard treatment of common illnesses of children in Papua New Guinea. 7 ed: Government of Papua New Guinea; 2000.

24. World Health Organization. Assessment and monitoring of antimalarial drug efficacy for the treatment of uncomplicated falciparum malaria. Geneva: World Health Organization; 2003.

25. White NJ, Stepniewska K, Barnes K, Price RN, Simpson J. Simplified antimalarial therapeutic monitoring: using the day-7 drug level? Trends Parasitol 2008;24:159-63.

26. Price RN, Hasugian AR, Ratcliff A, et al. Clinical and pharmacological determinants of the therapeutic response to dihydroartemisinin-piperaquine for drug-resistant malaria. Antimicrob Agents Chemother 2007;51:4090-7.

27. Basco LK, Ndounga M, Keundjian A, Ringwald P. Molecular epidemiology of malaria in cameroon. IX. Characteristics of recrudescent and persistent Plasmodium falciparum infections after chloroquine or amodiaquine treatment in children. Am J Trop Med Hyg 2002;66:117-23.

28. Sim IK, Davis TME, Ilett KF. Effects of a high-fat meal on the relative oral bioavailability of piperaquine. Antimicrobial Agents and Chemotherapy 2005;49:2407-2411.

29. Karl S, Davis TM, St-Pierre TG. A comparison of the sensitivities of detection of Plasmodium falciparum gametocytes by magnetic fractionation, thick blood film microscopy, and RT-PCR. Malar J 2009;8:98.

30. Trape JF. Rapid evaluation of malaria parasite density and standardization of thick smear examination for epidemiological investigations. Trans R Soc Trop Med Hyg 1985;79:181-4.

31. Tarning J, Lindegardh N, Annerberg A, et al. Pitfalls in estimating piperaquine elimination. Antimicrob Agents Chemother 2005;49:5127-8.

32. Butcher G, Sinden R. Persistence of atovaquone in human sera following treatment: inhibition of *Plasmodium falciparum* development in vivo and in vitro. Am J Trop Med Hyg 2003;68:111-4.
